# Efficient Solvent-Free Synthesis of Indolizines Using CuBr Catalyst from Pyridine, Acetophenone, and Electron-Deficient Alkenes

**DOI:** 10.3390/molecules29092061

**Published:** 2024-04-29

**Authors:** Xueguo Zhang, Jianpeng Zhang, Zhengyi Liu, Wenxuan Bi, Jian Shen, Guang Li

**Affiliations:** Department of Materials Science and Engineering, Liaocheng University, Liaocheng 252059, China

**Keywords:** Cu-catalyzed, oxidant, indolizine, solvent-free, green chemistry

## Abstract

Herein, we have developed a new approach for the synthesis of indolizine via Cu-catalyzed reaction of pyridine, acetophenone, and nitroolefin under mild conditions in high yields. This reaction involved the formation of C–N and C–C bonds and new indolizine compounds with high stereoselectivity and excellent functional group tolerance.

## 1. Introduction

Indolizine is a heterocyclic organic compound in many pharmaceuticals and natural products, characterized by a fused pyrrole and pyridine ring structure [1,2]. This unique structure gives it a wide range of chemical and biological properties, making it a valuable target for synthetic chemists [3,4]. The pharmaceutical industry has recognized the significance of indolizine-containing compounds and their potential as therapeutic agents. Researchers continue to explore the synthesis of novel indolizine derivatives with enhanced biological activities and improved pharmacokinetic properties [5].

In medicinal chemistry, indolizine derivatives have been found to exhibit significant biological activities, including anti-inflammatory, antimicrobial, antiviral, and anticancer properties [6]. This has led to a surge in research interest in this compound, with scientists seeking to understand its mechanisms of action and to develop new, more effective indolizine-based drugs [7,8,9]. In the field of materials science, indolizine has been studied for its potential use in the development of organic semiconductors and photovoltaic materials [10,11,12]. Its unique electronic properties, combined with its structural versatility, make it a promising candidate for more applications [13,14].

**Scheme 1:**
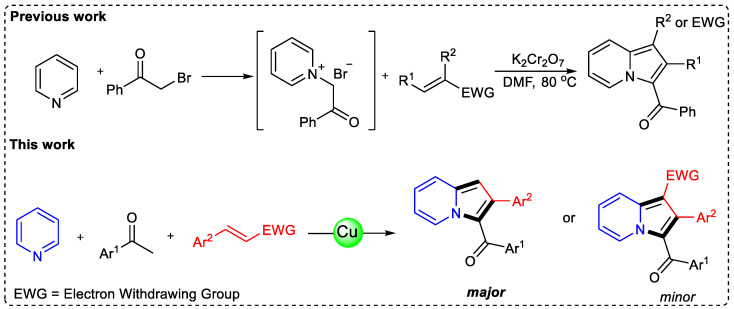
Synthesis of indolizines derivatives.

Synthetic chemists have developed numerous strategies to access indolizine derivatives. These methods involve the construction of the fused ring system through cyclization reactions, such as the Tschitschibabin reaction [15]. Additionally, functionalization of the indolizine core enables the introduction of diverse substituents, further expanding the scope of potential applications. Despite the numerous synthetic methods developed for indolizine derivatives, there remain certain hurdles to overcome [16,17,18,19]. For instance, the synthesis method reported by the Kan research group allows the reaction to occur under relatively mild temperature conditions but requires stepwise procedures (Scheme 1) [20]. Boruah’s group synthesized indolizines by using pyridine, bromoacetophenone, and alkyne under microwave conditions [21]. The aforementioned synthesis methods have established a solid foundation for the development and application of indolizine derivatives [22,23,24,25]. However, research on indolizine is still in its early stages. Many of its potential applications are yet to be fully explored, and the synthesis of new indolizine derivatives remains a challenging task. The growing interest in this compound and the promising results obtained so far suggest that indolizine will continue to be a significant focus of research in the coming years [26,27,28].

## 2. Results and Discussions

Encouraged by these promising results, we chose pyridine **1a** (0.4 mmol), acetophenone **2a** (0.4 mmol), and (*E*)-1-methyl-4-(2-nitrovinyl)benzene **3a** (0.2 mmol) as model substrates for the synthesis of indolizines in the presence of CuBr as a catalyst and PIDA as an oxidant. Disappointingly, we detected two new products, which were identified as indolizines compounds **4a** and **4a’** after analysis (Table 1**, entry 1** and Supplementary Material), and their yields were similar. Unfortunately, the extensive use of nitro compounds has led to the environmental contamination of soil and groundwater [29]. Subsequently, we adjusted the reaction conditions to increase the yield and selectivity of product **4a**. Initially, various copper salts were evaluated for this reaction. CuBr still exhibited the highest catalytic efficiency (Table 1**, entries 2**–**4)**. Next, we examined various types of oxidants separately. (NH_4_)_2_S_2_O_8_ was proved to be the best oxidant for this process [30]. (NH_4_)_2_S_2_O_8_ can provide the best oxidation effect when it is maintained at 1 equivalent, but if it exceeds 1 equivalent, the reaction yield will decrease. It was good to see that the yield was not just high, but the effect of denitrification was also noteworthy in the presence of CuBr and (NH_4_)_2_S_2_O_8_ (Table 1**, entries 5**–**10).** Simultaneously, it was established that the reaction temperature and time for this reaction were 130 °C and 5 h, respectively (Table 1**, entries 11**–**18**). Finally, using MeCN and DMF as reaction solvents respectively, the yield of the target product was significantly reduced (Table 1**, entries 19**–**20**). Therefore, we insisted on conducting the reaction in a solvent-free state. This approach not only enhances the reaction rate but also aligns with the principles of “green chemistry”. Through the screening of reaction conditions, we achieved a satisfactory yield of product **4a** using CuBr as the catalyst and (NH_4_)_2_S_2_O_8_ as an oxidant under solvent-free conditions at 130 °C for 5 h (Table 1**, entry 17**).

**Figure 1:**
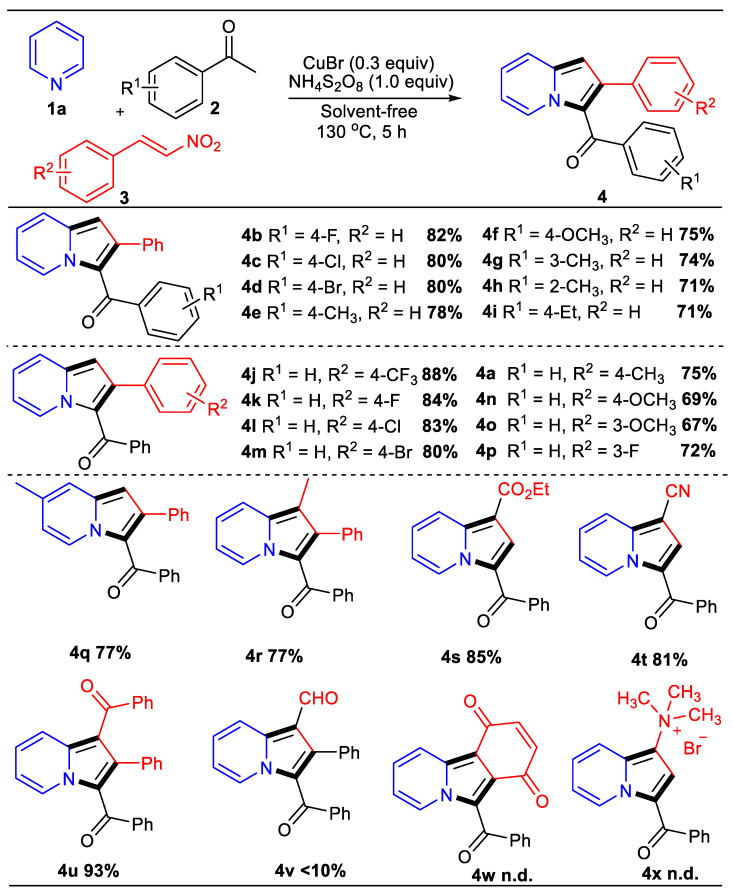
Scope of electron-deficient alkenes ^a,b^. ^a^ Reaction conditions: **1** (0.8 mmol), **2** (0.4 mmol), **3a** (0.2 mmol), CuBr, (NH_4_)_2_S_2_O_8_, sealed tube, at 130 °C in an oil bath for 5 h, unless otherwise specified. ^b^ Isolated yields. n.d.: not detected.

With the optimized conditions in hand, we turned to investigate the indolizines by using a systematic variation of acetophenones, pyridine **1a**, and (*E*)-(2-nitrovinyl)benzene **3b,** as shown in Figure 1. From the reaction results, it can be seen that the stronger the electron-withdrawing group of acetophenone, the easier it is to obtain a high-yield product (Figure 1, **4b**–**4d**). When acetophenone was substituted by electron-donating groups methyl, methoxy, and ethyl, the yield decreased slightly (Figure 1, **4e**, **4f**, and **4i**). At the same time, it can also be concluded that the steric hindrance effect of acetophenone has little impact on the reaction (Figure 1, **4g** and **4h**). After exploring the effect of acetophenone on the reaction, we turned to nitrostyrene. Through these reactions (Figure 1, **4a** and **4j**–**4p**), we reached a similar conclusion to the previous one: substrates with electron-withdrawing groups are more likely to promote the progression. In addition, we also investigated the reaction of 4-methyl-substituted pyridine with acetophenone and *(E)*-(2-nitrovinyl)benzene under standard conditions, and obtained the target product at a yield of 77% (Figure 1, **4q**). Finally, we also investigated other electron-deficient alkenes, such as 1-phenyl-2-nitropropene, ethyl acrylate, acrylonitrile, and chalcone, all of which achieved high target yields (Figure 1, **4r**–**4u**). What surprised us was that the reaction yields of two compounds with similar structures, chalcone and cinnamaldehyde, were very different (Figure 1, **4u** and **4v**). Unfortunately, the target product was not obtained when 1,4-benzoquinone, and trimethylvinylammonium bromide participated in the reaction system (Figure 1, **4w** and **4x**). This also demonstrates that although the reaction possesses excellent yield and selectivity, it has limitations. The limitation provides direction and theoretical guidance for us to continue to explore this type of reaction.

**Scheme 2:**
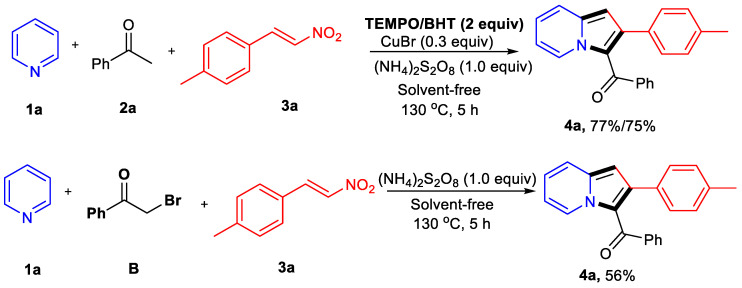
Control experiments.

To understand the reaction process, we carried out a few control experiments. Firstly, we added TEMPO/BHT into the reaction system as radical scavenger under standard conditions (Scheme 2). Luckily, we still managed to produce the desired product **4a**. This suggests that the reaction did not include a free radical process. In addition, we introduced *ω*-bromoacetophenone **B** into the reaction system with no CuBr catalyst. In this case, we also successfully obtained product **4a** (Scheme 2). This indicates that compound **B** is likely to be an intermediate structure in this transformation process. These experiments offered proof for the verification of the reaction mechanism.

**Scheme 3:**
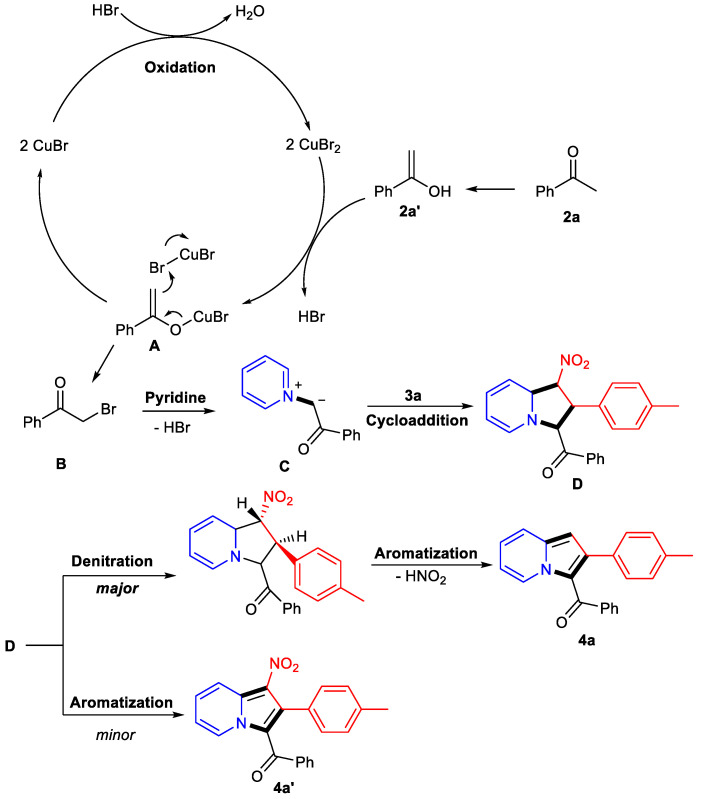
Proposed mechanism.

Based on these preliminary experimental results and literature precedents [31,32], we propose a plausible mechanism as shown in Scheme 3. Acetophenone **2a** is transformed into **2a’** through enolization, and then it reacts with one molecule of CuBr_2_ to produce an intermediate **A**, with HBr as a byproduct. The electron-rich intermediate **A** interacts with another molecule of CuBr_2_, leading to the formation of the product *ω*-bromoacetophenone **B**. Simultaneously, CuBr would be formed by getting rid of copper ions, which completed the catalytic cycle. *ω*-Bromoacetophenone **B** interacts with pyridine to produce an *N*-ylide intermediate **C**. The intermediate **C** reacts with **3a** in 1,3-dipolar cycloaddition process to produce intermediate **D**. Finally, the intermediate compound **D** is subjected to processes of denitration and aromatization to yield the product **4a [33,34]**. Additionally, intermediate **D** can also be directly converted into product **4a’** through the process of aromatization.

## 3. Materials and Methods

### 3.1. Materials

Pyridine, acetophenone, (*E*)-1-methyl-4-(2-nitrovinyl) benzene, and other raw materials were purchased from Bide Pharmatech Co., Ltd. (Shanghai, China) All commercially available organic and inorganic compounds were used directly without further purification.

### 3.2. Methods

#### 3.2.1. Test Methods

^1^H NMR spectra were recorded at 400 MHz or 500 MHz in CDCl_3_ and ^13^C NMR spectra were recorded on 101 MHz or 126 MHz in CDCl_3_, using TMS as the internal standard. The chemical shifts (δ) were measured in ppm and with the solvents as references (for CDCl_3_, ^1^H: δ = 7.26 ppm, ^13^C: δ = 77.0 ppm). All compounds were further characterized by HRMS; copies of their ^1^H NMR and ^13^C NMR spectra are provided. Products were purified by flash chromatography on 200–300 mesh silica gels. All melting points were determined on a microscopic apparatus without correction.

#### 3.2.2. Synthesis Methods

The general procedure for the synthesis of phenyl(2-(p-tolyl)indolizin-3-yl)methanone **4a** and (1-nitro-2-(p-tolyl)indolizin-3-yl)(phenyl)methanone **4a’** was as follows:

**1a** (0.8 mmol), **2a** (0.4 mmol), **3a** (0.2 mmol), (NH_4_)_2_S_2_O_8_ (1.0 equiv), and CuBr (0.3 equiv) were heated at 130 °C for 5 h in a sealed tube. When the reaction was completed (detected by TLC), the mixture was cooled to room temperature. The product **4a** and **4a’** was purified by column chromatography on silica gel (petroleum ether/EtOAc = 6:1).

phenyl(2-(p-tolyl)indolizin-3-yl)methanone (4a) yellow solid; mp 109–110 °C

^1^H NMR (500 MHz, CDCl_3_) δ 9.79 (d, J = 7.1 Hz, 1H), 7.53 (d, J = 8.8 Hz, 1H), 7.41 (dd, J = 8.1, 1.1 Hz, 2H), 7.15 (dd, J = 10.9, 4.8 Hz, 2H), 7.01 (t, J = 7.7 Hz, 2H), 6.96 (d, J = 8.0 Hz, 2H), 6.88 (td, J = 7.0, 1.3 Hz, 1H), 6.81 (d, J = 7.9 Hz, 2H), 6.56 (s, 1H), 2.20 (s, 3H). ^13^C NMR (126 MHz, CDCl_3_) δ 186.77, 140.15, 139.80, 137.57, 136.23, 132.88, 130.38, 129.90, 129.56, 128.21, 128.14, 127.29, 124.03, 120.01, 118.25, 113.34, 103.99, 20.97. HRMS (ESI): *m/z* calcd for C_22_H_18_NO (M + H)^+^ 312.1388; found: 312.1385.

(1-nitro-2-(p-tolyl)indolizin-3-yl)(phenyl)methanone (4a’) yellow solid; mp 138–140 °C

^1^H NMR (500 MHz, CDCl_3_) δ 9.53–9.40 (m, 1H), 8.65 (dt, *J* = 9.1, 1.1 Hz, 1H), 7.64 (ddd, *J* = 9.0, 6.9, 1.0 Hz, 1H), 7.38 (dd, *J* = 8.2, 1.2 Hz, 2H), 7.24–7.20 (m, 1H), 7.17 (td, *J* = 7.0, 1.4 Hz, 1H), 7.09–7.00 (m, 4H), 6.84 (d, *J* = 7.8 Hz, 2H), 2.18 (s, 3H). ^13^C NMR (126 MHz, CDCl_3_) δ 188.49, 138.23, 137.99, 134.62, 134.16, 131.58, 130.94, 130.19, 129.21, 128.10, 128.06, 127.70, 127.62, 121.39, 119.34, 116.30, 21.09. HRMS (ESI): *m/z* calcd for C_22_H_17_N_2_O_3_ (M + H)^+^ 357.1239; found: 357.1236.

## 4. Conclusions

In conclusion, the reaction of pyridine, acetophenone, and nitroolefin under the catalysis of CuBr and (NH_4_)_2_S_2_O_8_ as the oxidant is a crucial process in the synthesis of indolizine. This reaction showcases the importance of catalysts and oxidants in facilitating chemical transformations and the production of valuable organic compounds. At the same time, the reaction occurs under solvent-free conditions, reflecting environmental friendliness. Further studies on the mechanistic details and synthetic applications of this method are in progress in our group. It is believed that with the progress of science and technology, more potential applications of indolizine will be found.

## Figures and Tables

**Table 1:** Optimization of reaction conditions ^a^.

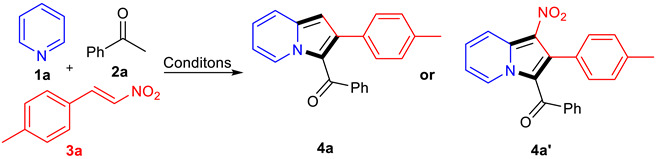					
Entry	Cat.	Oxidant (x Equiv)	*T* (°C)	*t* (h)	Yield [%] ^b^
					4a (4a’)
1	CuBr	PIDA (2.0)	110	12	34 (30)
2	CuCl	PIDA (2.0)	110	12	26 (20)
3	Cu_2_O	PIDA (2.0)	110	12	24 (22)
4	Cu_2_S	PIDA (2.0)	110	12	24 (25)
5	CuBr	O_2_	110	12	36 (30)
6	CuBr	IBX (2.0)	110	12	35 (31)
7	CuBr	(NH_4_)_2_S_2_O_8_ (2.0)	110	12	65 (14)
8	CuBr	(NH_4_)_2_S_2_O_8_ (1.0)	110	12	71 (15)
9	CuBr	(NH_4_)_2_S_2_O_8_ (3.0)	110	12	63
10	CuBr	(NH_4_)_2_S_2_O_8_ (4.0)	110	12	56
11	CuBr	(NH_4_)_2_S_2_O_8_ (1.0)	120	12	72
12	CuBr	(NH_4_)_2_S_2_O_8_ (1.0)	130	12	76
13	CuBr	(NH_4_)_2_S_2_O_8_ (1.0)	140	12	73
14	CuBr	(NH_4_)_2_S_2_O_8_ (1.0)	130	10	75
15	CuBr	(NH_4_)_2_S_2_O_8_ (1.0)	130	8	76
16	CuBr	(NH_4_)_2_S_2_O_8_ (1.0)	130	6	78
17	CuBr	(NH_4_)_2_S_2_O_8_ (1.0)	130	5	80 (<5)
18	CuBr	(NH_4_)_2_S_2_O_8_ (1.0)	130	4	78
19 ^c^	CuBr	(NH_4_)_2_S_2_O_8_ (1.0)	130	5	76
20 ^d^	CuBr	(NH_4_)_2_S_2_O_8_ (1.0)	130	5	55

^a^ Reaction conditions: **1a** (0.4 mmol), **2a** (0.4 mmol), **3a** (0.2 mmol), copper catalyst (0.3 equiv), oxidant, solvent-free sealed tube in an oil bath, unless otherwise specified. ^b^ Isolated yields. ^c^ Reacting solvent was MeCN. ^d^ Reacting solvent was DMF. PID: phenyliodine(III) diacetate. IBX: 2-Iodoxybenzoic acid. DMF: *N*,*N*- dimethylformamide.

## Data Availability

Data are contained within the article and Supplementary Materials.

## Supplementary Materials

The following supporting information can be downloaded at https://www.mdpi.com/article/10.3390/molecules29092061/s1. NMR spectra, melting points, and HRMS of (**4a**–**4u**) detailed data are available as Supplementary Materials.
