# Supplementary material for: Efficient Solvent-Free Synthesis of Indolizines Using CuBr Catalyst from Pyridine, Acetophenone, and Electron-Deficient Alkenes
Source: Molecules. 2024 Apr 29;29(9):2061. doi: 10.3390/molecules29092061 (PMC11085153; doi:10.3390/molecules29092061)

Supplementary Material

Efficient Solvent-Free Synthesis of Indolizines Using CuBr Catalyst from Pyridine, Acetophenone, and Electron-Deficient Alkenes

Xueguo Zhang *, Jianpeng Zhang, Zhengyi Liu, Wenxuan Bi, Jian Shen and Guang Li *

Department of Materials Science and Engineering, Liaocheng University, Liaocheng 252059, China;

* Correspondence: zhangxueguo@lcu.edu.cn (X.Z.); lglzsd@126.com (G.L.)

1. General remark

^1H NMR spectra were recorded at 400 MHz or 500 MHz in $CDCl_3$, ^{13}C NMR spectra were recorded on 101 MHz or 126 MHz in $CDCl_3$, with using TMS as internal standard. The chemical shifts (δ) were measured in ppm and with the solvents as references (For $CDCl_3$, ^1H: δ = 7.26 ppm, ^{13}C: δ = 77.0 ppm). All compounds were further characterized by HRMS; copies of their ^1H NMR and ^{13}C NMR spectra are provided. Products were purified by flash chromatogrgraphy on 200-300 mesh silica gels. All melting points were determined on a microscopic apparatus without correction. Unless otherwise noted, commercially reagents and were used without further purification.

2. General procedure for the synthesis of phenyl(2-(p-tolyl)indolizin-3-yl)methanone **4a** and (1-nitro-2-(p-tolyl)indolizin-3-yl)(phenyl)methanone **4a'**

1a (0.8 mmol), **2a** (0.4 mmol), **3a** (0.2 mmol), $(NH_4)_2S_2O_8$ (1.0 equiv) and CuBr (0.3 equiv), were heated at 130 °C for 5 h in a sealed tube. When the reaction was completed (detected by TLC), the mixture was cooled to room temperature. The product **4a** and **4a'** was purified by column chromatography on silica gel (petroleum ether/EtOAc = 6:1).

3. Compounds Characterization Data

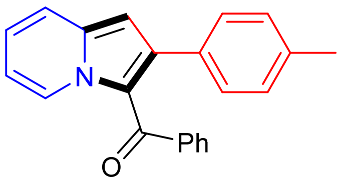

4a

phenyl(2-(p-tolyl)indolizin-3-yl)methanone (4a) Yellow solid; mp 109-110 °C

^{1}H NMR (500 MHz, $CDCl_3$) δ 9.79 (d, J = 7.1 Hz, 1H), 7.53 (d, J = 8.8 Hz, 1H), 7.41 (dd, J = 8.1, 1.1 Hz, 2H), 7.15 (dd, J = 10.9, 4.8 Hz, 2H), 7.01 (t, J = 7.7 Hz, 2H), 6.96 (d, J = 8.0 Hz, 2H), 6.88 (td, J = 7.0, 1.3 Hz, 1H), 6.81 (d, J = 7.9 Hz, 2H), 6.56 (s, 1H), 2.20 (s, 3H). ^{13}C NMR (126 MHz, $CDCl_3$) δ 186.77, 140.15, 139.80, 137.57, 136.23, 132.88, 130.38, 129.90, 129.56, 128.21, 128.14, 127.29, 124.03, 120.01, 118.25, 113.34, 103.99, 20.97. HRMS (ESI): m/z calcd for $C_{22}H_{18}NO$ $(M + H)^+$ 312.1388; found: 312.1385.

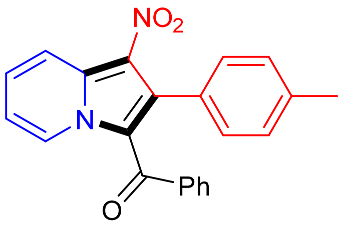

H

4a'

(1-nitro-2-(p-tolyl)indolizin-3-yl)(phenyl)methanone (4a') Yellow solid; mp 138-140 °C

^{1}H NMR (500 MHz, $CDCl_3$) δ 9.53 – 9.40 (m, 1H), 8.65 (dt, *J* = 9.1, 1.1 Hz, 1H), 7.64 (ddd, *J* = 9.0, 6.9, 1.0 Hz, 1H), 7.38 (dd, *J* = 8.2, 1.2 Hz, 2H), 7.24 – 7.20 (m, 1H), 7.17 (td, *J* = 7.0, 1.4 Hz, 1H), 7.09 – 7.00 (m, 4H), 6.84 (d, *J* = 7.8 Hz, 2H), 2.18 (s, 3H). ^{13}C NMR (126 MHz, $CDCl_3$) δ 188.49, 138.23, 137.99, 134.62, 134.16, 131.58, 130.94, 130.19, 129.21, 128.10, 128.06, 127.70, 127.62, 121.39, 119.34, 116.30, 21.09. HRMS (ESI): m/z calcd for $C_{22}H_{17}N_2O_3$ $(M + H)^+$ 357.1239; found: 357.1236.

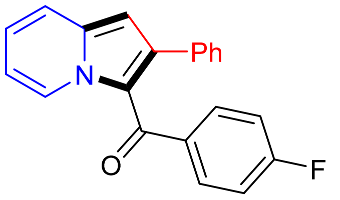

4b

(4-fluorophenyl)(2-phenylindolizin-3-yl)methanone (4b) Yellow solid; mp 106-109 °C

^{1}H NMR (500 MHz, $CDCl_3$) δ 9.76 (d, J = 7.2 Hz, 1H), 7.55 (d, J = 8.8 Hz, 1H), 7.42 (dd, J = 8.7, 5.5 Hz, 2H), 7.18 (ddd, J = 8.7, 6.8, 0.9 Hz, 1H), 7.10 – 7.03 (m, 5H), 6.90 (td, J = 7.1, 1.2 Hz, 1H), 6.68 (t, J = 8.7 Hz, 2H), 6.59 (s, 1H). ^{13}C NMR (126 MHz, $CDCl_3$) δ 185.14, 165.10, 163.10, 139.65, 137.66, 136.36, 136.34, 135.76, 131.87, 131.80, 131.12, 130.08, 128.11, 127.66, 127.55, 126.78, 124.26, 119.84, 118.37, 114.42, 114.24, 113.59, 104.18, ^{19}F NMR (471 MHz, $CDCl_3$) δ -109.12. HRMS (ESI): m/z calcd for $C_{21}H_{15}FNO$ $(M + H)^+$ 316.1138; found: 316.1135.

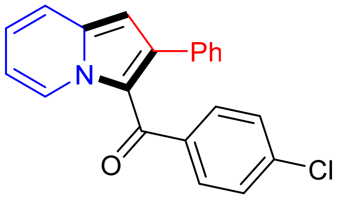

4c

(4-chlorophenyl)(2-phenylindolizin-3-yl)methanone (4c) Yellow solid; mp 110-113 °C

^{1}H NMR (500 MHz, $CDCl_3$) δ 9.80 (d, *J* = 7.0 Hz, 1H), 7.55 (d, *J* = 8.8 Hz, 1H), 7.32 (d, *J* = 8.4 Hz, 2H), 7.19 (dd, *J* = 11.2, 4.3 Hz, 1H), 7.09 – 7.02 (m, 5H), 6.98 – 6.94 (m, 2H), 6.91 (td, *J* = 7.1, 1.2 Hz, 1H), 6.59 (s, 1H). ^{13}C NMR (126 MHz, $CDCl_3$) δ 185.16, 139.89, 138.59, 137.83, 136.65, 135.70, 130.87, 130.10, 128.23, 127.72, 127.54, 126.82, 124.52, 118.40, 113.75, 104.36. HRMS (ESI): m/z calcd for $C_{21}H_{15}ClNO$ $(M + H)^+$ 332.0842; found: 332.0840.

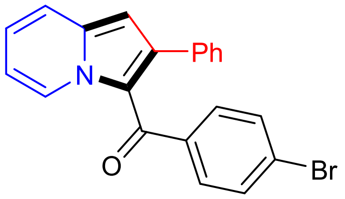

4d

(4-bromophenyl)(2-phenylindolizin-3-yl)methanone (4d) Yellow solid; mp 109-110 °C

1H NMR (500 MHz, $CDCl_3$) δ 9.73 (d, J = 6.9 Hz, 1H), 7.48 (d, J = 8.8 Hz, 1H), 7.17 (d, J = 8.4 Hz,

2H), 7.14 – 7.09 (m, 1H), 7.03 (dd, J = 12.4, 6.7 Hz, 3H), 6.97 (d, J = 4.3 Hz, 4H), 6.83 (td, J = 7.0, 1.1 Hz, 1H), 6.51 (s, 1H). 13C NMR (126 MHz, $CDCl_3$) δ 184.20, 138.91, 137.99, 136.82, 134.64, 129.97, 129.46, 129.06, 127.22, 126.69, 125.76, 124.13, 123.53, 118.81, 117.36, 112.74, 103.36. HRMS (ESI): m/z calcd for $C_{21}H_{15}BrNO$ $(M + H)^+$ 376.0337; found: 376.0339.

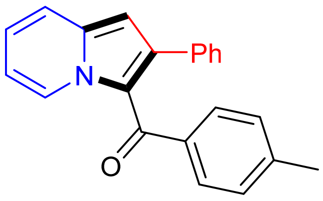

4e

(2-phenylindolizin-3-yl)(p-tolyl)methanone (4e) Yellow solid; mp 110-111 °C

^{1}H NMR (400 MHz, $CDCl_3$) δ 9.73 (d, J = 7.2 Hz, 1H), 7.53 (d, J = 8.8 Hz, 1H), 7.32 (d, J = 8.1 Hz, 2H), 7.16 – 7.11 (m, 1H), 7.05 (ddd, J = 6.8, 5.6, 1.8 Hz, 5H), 6.89 – 6.78 (m, 3H), 6.58 (s, 1H), 2.19 (s, 3H). ^{13}C NMR (101 MHz, $CDCl_3$) δ 186.72, 141.08, 139.36, 137.33, 136.01, 130.08, 129.76, 128.01, 127.53, 126.34, 123.78, 120.14, 118.34, 113.30, 103.83, 21.33. HRMS (ESI): m/z calcd for $C_{22}H_{18}NO$ $(M + H)^+$ 312.1388; found: 312.1386.

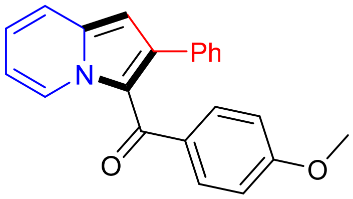

4f

(4-methoxyphenyl)(2-phenylindolizin-3-yl)methanone (4f) Yellow solid; mp 119-120 °C

^{1}H NMR (500 MHz, $CDCl_3$) δ 9.65 (d, *J* = 7.2 Hz, 1H), 7.53 (d, *J* = 8.8 Hz, 1H), 7.43 (d, *J* = 8.8 Hz, 2H), 7.15 – 7.10 (m, 3H), 7.08 – 7.03 (m, 3H), 6.85 (td, *J* = 7.0, 1.3 Hz, 1H), 6.59 (s, 1H), 6.54 – 6.50 (m, 2H), 3.70 (s, 3H) ^{13}C NMR (126 MHz, $CDCl_3$) δ 185.87, 161.83, 138.90, 137.21, 136.05, 132.61, 131.81, 130.12, 127.88, 127.67, 126.53, 123.54, 120.06, 118.38, 113.17, 112.74, 103.58, 55.28 (s). HRMS (ESI): m/z calcd for $C_{22}H_{18}NO_2$ $(M + H)^+$ 328.1338; found: 328.1336.

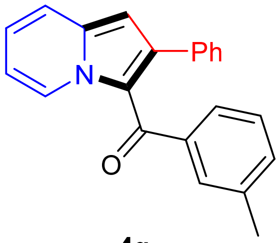

4g

(2-phenylindolizin-3-yl)(m-tolyl)methanone (4g) Yellow solid; mp 87-88 °C

^{1}H NMR (400 MHz, $CDCl_3$) δ 9.80 (d, J = 7.2 Hz, 4H), 7.54 (d, J = 8.8 Hz, 4H), 7.29 (dd, J = 6.1, 2.5 Hz, 4H), 7.16 (dd, J = 10.5, 4.9 Hz, 8H), 7.08 (dt, J = 6.2, 3.7 Hz, 10H), 7.04 – 6.99 (m, 12H), 6.94 (dd, J = 5.2, 4.4 Hz, 9H), 6.88 (td, J = 7.0, 1.3 Hz, 4H), 6.58 (s, 4H), 2.06 (s, 12H). ^{13}C NMR (101 MHz, $CDCl_3$) δ 186.91 (s), 139.83 (d, J = 16.7 Hz), 137.48, 136.86, 136.18, 131.33, 130.90, 130.68, 129.89, 128.18, 127.46, 127.32 126.57, 124.06, 120.14, 118.34, 113.48, 104.09, 20.90. HRMS (ESI): m/z calcd for $C_{22}H_{18}NO$ $(M + H)^+$ 312.1388; found: 312.1391

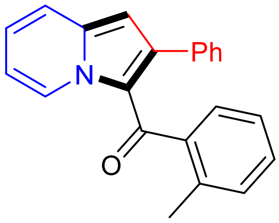

4h

(2-phenylindolizin-3-yl)(o-tolyl)methanone (4h) Yellow solid; mp 97-98 °C

^{1}H NMR (500 MHz, $CDCl_3$) δ 10.09 (d, J = 7.1 Hz, 1H), 7.55 (d, J = 8.8 Hz, 1H), 7.25 – 7.20 (m, 1H), 7.04 – 6.93 (m, 8H), 6.87 (d, J = 7.6 Hz, 1H), 6.72 (t, J = 7.4 Hz, 1H), 6.52 (s, 1H), 2.32 (s, 3H). ^{13}C NMR (126 MHz, $CDCl_3$) δ 187.63, 140.79, 140.56, 137.81, 135.86, 135.71, 130.03, 129.50, 129.16, 128.86, 127.16, 126.48, 124.76, 124.69, 120.74, 118.25, 113.89, 105.04, 19.68. HRMS (ESI): m/z calcd for $C_{22}H_{18}NO$ $(M + H)^+$ 312.1388; found: 312.1389

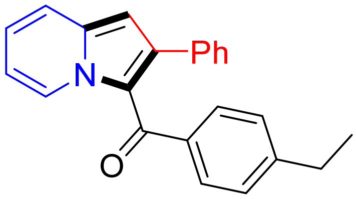

4i

(4-ethylphenyl)(2-phenylindolizin-3-yl)methanone (4i) Yellow solid; mp 86-88 °C

^{1}H NMR (500 MHz, $CDCl_3$) δ 9.77 (dd, *J* = 7.2, 0.6 Hz, 1H), 7.53 (d, *J* = 8.9 Hz, 1H), 7.33 (d, *J* = 8.1 Hz, 2H), 7.14 (ddd, *J* = 8.7, 6.7, 0.9 Hz, 1H), 7.08 – 7.04 (m, 2H), 7.02 – 6.95 (m, 3H), 6.87 (td, *J* = 7.1, 1.3 Hz, 1H), 6.81 (d, *J* = 8.1 Hz, 2H), 6.58 (s, 1H), 2.47 (q, *J* = 7.6 Hz, 2H), 1.09 (t, *J* = 7.6 Hz, 3H). ^{13}C NMR (126 MHz, $CDCl_3$) δ 186.76, 147.37, 139.57, 137.55, 136.05, 130.07, 129.81, 128.12, 127.50, 126.87, 126.44, 123.89, 120.19, 118.35, 113.37, 103.93, 28.83, 15.53. HRMS (ESI): m/z calcd for $C_{23}H_{20}NO$ $(M + H)^+$ 326.1545; found: 326.1546

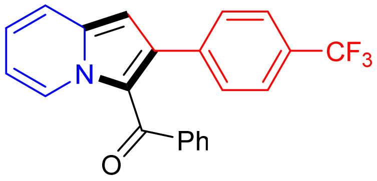

4j

phenyl(2-(4-(trifluoromethyl)phenyl)indolizin-3-yl)methanone (4j) Yellow solid; mp 62-63 °C

^{1}H NMR (500 MHz, $CDCl_3$) δ 9.83 (dd, *J* = 7.2, 0.6 Hz, 1H), 7.58 (d, *J* = 8.8 Hz, 1H), 7.36 (dd, *J* = 8.2, 1.2 Hz, 2H), 7.26 – 7.19 (m, 3H), 7.15 (ddd, *J* = 5.6, 4.5, 2.2 Hz, 3H), 7.00 (t, *J* = 7.8 Hz, 2H), 6.94 (td, *J* = 7.0, 1.3 Hz, 1H), 6.60 (s, 1H). ^{13}C NMR (126 MHz, $CDCl_3$) δ 186.51, 139.93, 139.76, 138.11, 137.62, 130.85, 130.20, 129.42, 128.24, 127.50, 124.54, (124.361, 124.33, 124.30, 124.27 (q, *J* = 3.9 Hz), 120.12, 118.51, 114.05, 104.22, ^{19}F NMR (471 MHz, $CDCl_3$) δ -62.77 (s). HRMS (ESI): m/z calcd for $C_{22}H_{15}F_3NO$ $(M + H)^+$ 366.1106; found: 366.1104

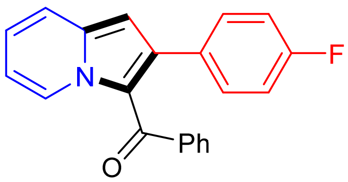

4k

(2-(4-fluorophenyl)indolizin-3-yl)(phenyl)methanone (4k) Yellow solid; mp 72-74 °C

^{1}H NMR (400 MHz, $CDCl_3$) δ 9.73 (dd, *J* = 7.2, 0.7 Hz, 1H), 7.47 (d, *J* = 8.8 Hz, 1H), 7.35 – 7.29 (m, 2H), 7.14 – 7.08 (m, 2H), 6.99 – 6.93 (m, 4H), 6.83 (td, *J* = 7.0, 1.4 Hz, 1H), 6.65 – 6.59 (m, 2H), 6.47 (s, 1H). ^{13}C NMR (101 MHz, $CDCl_3$) δ 186.61, 162.95, 160.50, 140.04 , 138.59, 137.58, 131.46,130.76,

129.54, 128.19, 127.46, 124.34, 118.33, 114.60, 114.39, 113.67, 104.11, ^{19}F NMR (376 MHz, $CDCl_3$) δ -115.75 (s). HRMS (ESI): m/z calcd for $C_{21}H_{15}FNO$ $(M + H)^+$ 316.1138; found: 316.1134.

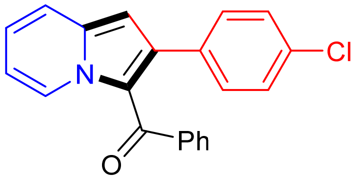

4l

(2-(4-chlorophenyl)indolizin-3-yl)(phenyl)methanone (4l) Yellow solid; mp 175-176 °C

^{1}H NMR (500 MHz, $CDCl_3$) δ 9.80 (d, *J* = 7.1 Hz, 1H), 7.54 (d, *J* = 8.8 Hz, 1H), 7.39 (d, *J* = 7.1 Hz, 2H), 7.20 (dt, *J* = 15.9, 7.7 Hz, 2H), 7.07 – 6.97 (m, 6H), 6.94 – 6.87 (m, 1H), 6.55 (s, 1H). ^{13}C NMR (126 MHz, $CDCl_3$) δ 186.57, 140.02, 138.33, 137.58, 134.48, 132.71, 131.16, 130.79, 129.53, 128.18, 127.68, 127.52, 124.34, 120.00, 118.38, 113.75, 104.05, HRMS (ESI): m/z calcd for $C_{21}H_{15}ClNO$ $(M + H)^+$ 332.0842; found: 332.0840.

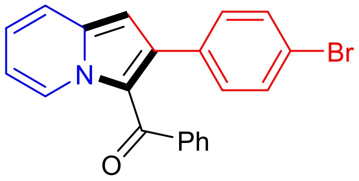

4m

(2-(4-bromophenyl)indolizin-3-yl)(phenyl)methanone (4m) Yellow solid; mp 149-150 °C

^{1}H NMR (400 MHz, $CDCl_3$) δ 9.80 (d, *J* = 7.2 Hz, 1H), 7.55 (d, *J* = 8.8 Hz, 1H), 7.44 – 7.33 (m, 2H), 7.23 – 7.16 (m, 2H), 7.15 – 7.08 (m, 2H), 7.05 (t, *J* = 7.7 Hz, 2H), 6.94 – 6.89 (m, 3H), 6.55 (s, 1H). ^{13}C NMR (101 MHz, $CDCl_3$) δ 186.56, 139.99, 138.33, 137.59, 134.94, 131.48, 130.80, 130.63, 129.53, 128.19, 127.55, 124.38, 120.91, 118.40, 113.79, 104.01. HRMS (ESI): m/z calcd for $C_{21}H_{15}BrNO$ $(M + H)^+$ 376.0337; found: 376.0334.

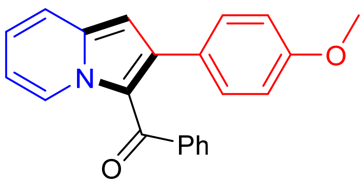

4n

(2-(4-methoxyphenyl)indolizin-3-yl)(phenyl)methanone (4n) Yellow solid; mp 139-140 °C

^{1}H NMR (400 MHz, $CDCl_3$) δ 10.06 (d, J = 7.1 Hz, 1H), 7.78 (d, J = 8.8 Hz, 1H), 7.72 – 7.64 (m, 2H), 7.45 – 7.38 (m, 2H), 7.31 – 7.24 (m, 4H), 7.13 (td, J = 7.0, 1.3 Hz, 1H), 6.81 (d, J = 8.4 Hz, 3H), 3.95 (s, 3H). ^{13}C NMR (126 MHz, $CDCl_3$) δ 186.75, 158.44, 140.14, 139.51, 137.64, 132.31, 131.14, 130.57, 129.60, 129.30, 128.36, 128.18, 127.39, 124.14, 120.01, 118.22, 113.34, 113.18, 103.89, 55.25. HRMS (ESI): m/z calcd for $C_{22}H_{18}NO_2$ $(M + H)^+$ 328.1338; found: 328.1339.

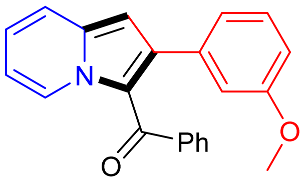

4o

(2-(3-methoxyphenyl)indolizin-3-yl)(phenyl)methanone (4o) Yellow solid; mp 135-138 °C

^{1}H NMR (500 MHz, $CDCl_3$) δ 9.81 (d, J = 7.1 Hz, 1H), 7.53 (d, J = 8.8 Hz, 1H), 7.47 – 7.43 (m, 2H), 7.16 (dt, J = 8.4, 3.9 Hz, 2H), 7.04 (t, J = 7.7 Hz, 2H), 6.96 (t, J = 7.8 Hz, 1H), 6.88 (tt, J = 9.6, 4.8 Hz, 1H), 6.74 (d, J = 7.6 Hz, 1H), 6.61 – 6.53 (m, 3H), 3.59 (s, 3H). ^{13}C NMR (126 MHz, $CDCl_3$) δ 186.68, 158.73, 140.28, 139.63, 137.57, 137.29, 130.74, 129.49, 128.68, 128.19, 127.40, 124.26, 122.57, 120.01, 118.40, 115.37, 113.64, 113.04, 104.24, 55.08. HRMS (ESI): m/z calcd for $C_{22}H_{18}NO_2$ $(M + H)^+$ 328.1338; found: 328.1341

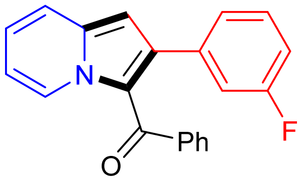

4p

(2-(3-fluorophenyl)indolizin-3-yl)(phenyl)methanone (4p) Yellow solid; mp 124-126 °C

^1H NMR (500 MHz, $CDCl_3$) δ 9.80 (d, J = 7.2 Hz, 1H), 7.55 (d, J = 8.8 Hz, 1H), 7.42 (d, J = 7.2 Hz, 2H), 7.19 (t, J = 7.5 Hz, 2H), 7.05 (t, J = 7.6 Hz, 2H), 6.93 (ddd, J = 21.7, 10.5, 4.4 Hz, 2H), 6.85 – 6.77 (m, 2H), 6.71 (td, J = 8.4, 2.2 Hz, 1H), 6.57 (s, 1H). ^{13}C NMR (126 MHz, $CDCl_3$) δ 186.61, 163.03, 161.08, 140.14, 138.26, 138.25, 138.22, 137.50, 130.85, 129.43, 129.43, 129.02, 128.16, 127.48, 125.93, 124.35, 119.98, 118.46, 116.87, 116.69, 113.85, 113.53, 113.36, 104.14. ^{19}F NMR (376 MHz, $CDCl_3$) δ -114.56 (s). HRMS (ESI): m/z calcd for $C_{21}H_{15}FNO$ $(M + H)^+$ 316.1138; found: 316.1137.

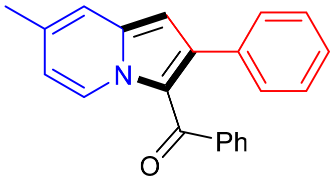

4q

(7-methyl-2-phenylindolizin-3-yl)(phenyl)methanone (4q) Yellow solid; mp 106-108 °C

^1H NMR (500 MHz, $CDCl_3$) δ 9.75 (d, J = 7.2 Hz, 1H), 7.39 (d, J = 7.2 Hz, 2H), 7.31 (s, 1H), 7.12 (t, J = 7.4 Hz, 1H), 7.06 (dd, J = 6.5, 2.9 Hz, 2H), 6.99 (dd, J = 8.7, 4.8 Hz, 5H), 6.75 (dd, J = 7.2, 1.5 Hz, 1H), 6.45 (s, 1H), 2.42 (s, 3H). ^{13}C NMR (126 MHz, $CDCl_3$) δ 186.21 (s), 140.35, 140.29, 138.19, 136.12, 135.33, 130.37, 130.04, 129.53, 127.82, 127.47, 127.27, 126.50, 119.65, 116.86, 116.15, 103.28, 21.26. HRMS (ESI): m/z calcd for $C_{22}H_{18}NO$ $(M + H)^+$ 312.1388; found: 312.1384

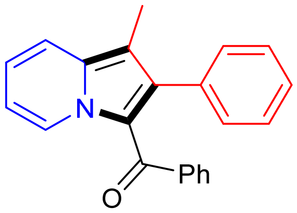

4r

(1-methyl-2-phenylindolizin-3-yl)(phenyl)methanone (4r) Yellow solid; mp 132-134 °C

^1H NMR (500 MHz, $CDCl_3$) δ 9.87 (d, J = 7.1 Hz, 1H), 7.52 (d, J = 8.8 Hz, 1H), 7.32 (d, J = 7.0 Hz, 2H), 7.17 (ddd, J = 8.7, 6.8, 0.8 Hz, 1H), 7.08 (t, J = 7.4 Hz, 1H), 7.04 – 6.92 (m, 7H), 6.88 (td, J = 7.0, 1.3 Hz, 1H), 2.23 (s, 3H). ^{13}C NMR (126 MHz, $CDCl_3$) δ 186.30, 140.42, 137.79, 136.58, 134.93, 131.00, 130.07, 129.22, 128.29, 127.36, 127.20, 126.42, 123.59, 120.27, 116.79, 113.62, 110.72, 9.20.

HRMS (ESI): m/z calcd for $C_{22}H_{18}NO$ $(M + H)^+$ 312.1388; found: 312.1391

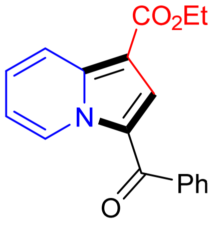

4s

ethyl 3-benzoylindolizine-1-carboxylate (4s) Yellow solid; mp 75-76 °C

^{1}H NMR (500 MHz, $CDCl_3$) δ 9.97 (d, J = 7.0 Hz, 1H), 8.40 (d, J = 8.9 Hz, 1H), 7.82 (dd, J = 6.3, 2.0 Hz, 3H), 7.60 – 7.55 (m, 1H), 7.52 (t, J = 7.4 Hz, 2H), 7.45 (ddd, J = 8.8, 6.9, 1.0 Hz, 1H), 7.09 (td, J = 7.0, 1.2 Hz, 1H), 4.38 (q, J = 7.1 Hz, 2H), 1.40 (t, J = 7.1 Hz, 3H). ^{13}C NMR (126 MHz, $CDCl_3$) δ 185.59, 164.08, 139.93, 139.90, 131.47, 129.02, 128.97, 128.40, 127.69, 122.54, 119.52, 115.29, 106.31, 60.11, 14.55. HRMS (ESI): m/z calcd for $C_{18}H_{16}NO_3$ $(M + H)^+$ 294.1130; found: 294.1132

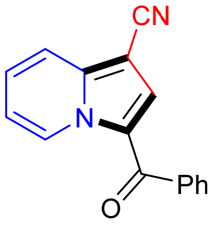

4t

3-benzoylindolizine-1-carbonitrile (4t) Yellow solid; mp 113-114 °C

^{1}H NMR (500 MHz, $CDCl_3$) δ 9.87 (d, J = 7.1 Hz, 1H), 7.73 (dd, J = 19.6, 8.2 Hz, 3H), 7.57 – 7.49 (m, 2H), 7.48 – 7.38 (m, 3H), 7.07 (t, J = 7.0 Hz, 1H). ^{13}C NMR (126 MHz, $CDCl_3$) δ 185.16, 141.25, 139.22, 132.00, 129.52, 129.27, 128.92, 128.87 , 128.56, 127.88, 127.76, 122.93, 117.52, 115.89, 115.13, 84.85. HRMS (ESI): m/z calcd for $C_{16}H_{11}N_2O$ $(M + H)^+$ 247.0871; found: 247.0874.

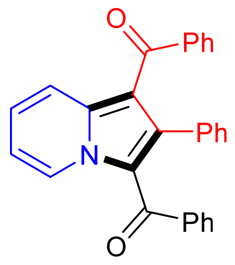

4u

(2-phenylindolizine-1,3-diyl)bis(phenylmethanone): Yellow solid; mp 184-186 ºC.

^{1}H NMR (500 MHz, $CDCl_3$) δ 9.68 (d, *J* = 7.1 Hz, 1H), 8.19 (d, *J* = 8.9 Hz, 1H), 7.48 – 7.31 (m, 5H), 7.17 (t, *J* = 7.4 Hz, 1H), 7.13 – 7.05 (m, 2H), 7.03 (t, *J* = 7.7 Hz, 2H), 6.96 (t, *J* = 7.7 Hz, 2H), 6.87 – 6.81 (m, 2H), 6.75 – 6.66 (m, 3H). ^{13}C NMR (126 MHz, $CDCl_3$) δ 192.92 (s), 188.44 (s), 139.14, 133.32, 131.64, 131.46, 131.23, 129.43, 129.41, 127.75, 127.53, 127.44, 127.24, 127.06, 126.91, 121.13, 119.20, 115.30, 114.08. HRMS (ESI): m/z calcd for $C_{28}H_{20}NO_2$ $(M + H)^+$ 402.1489; found: 402.1483

4. ^{1}H and ^{13}C NMR spectra of the products

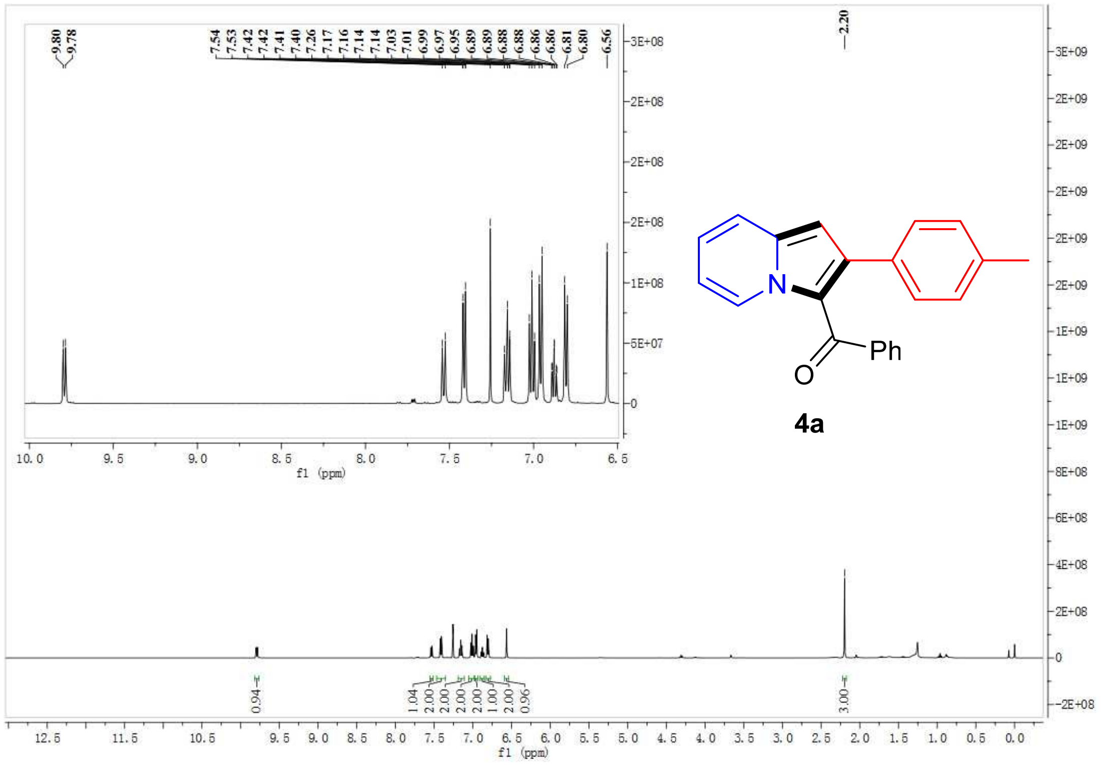

4a

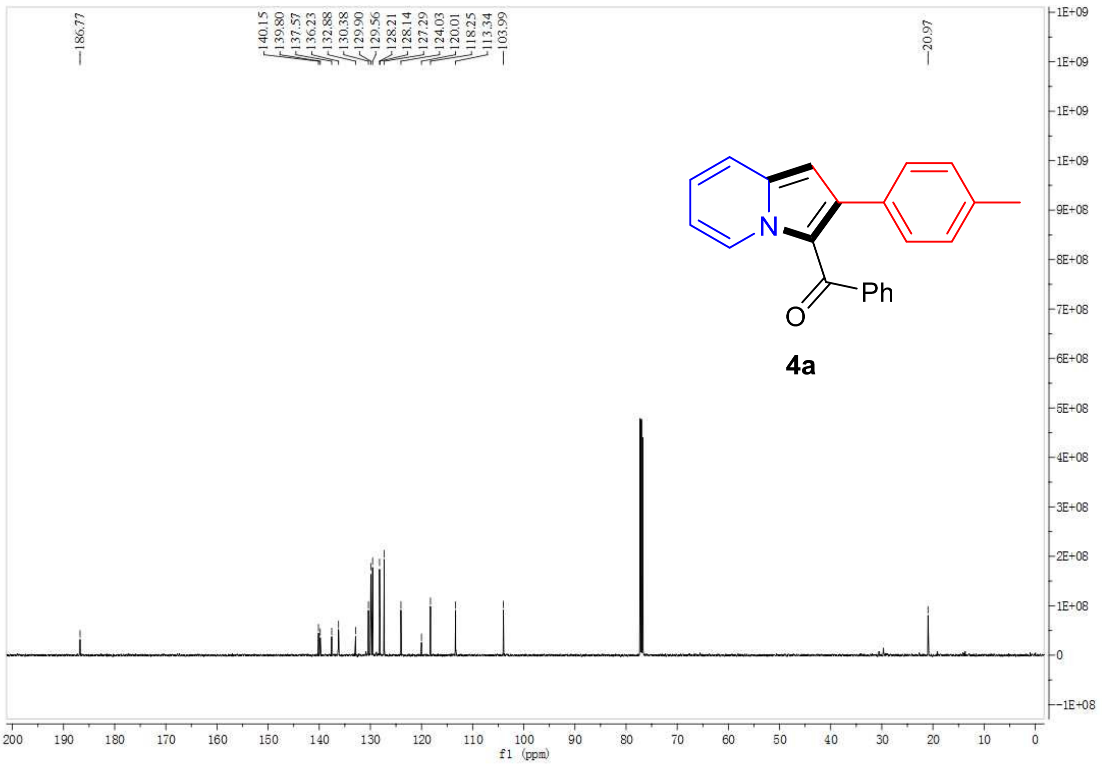

4a

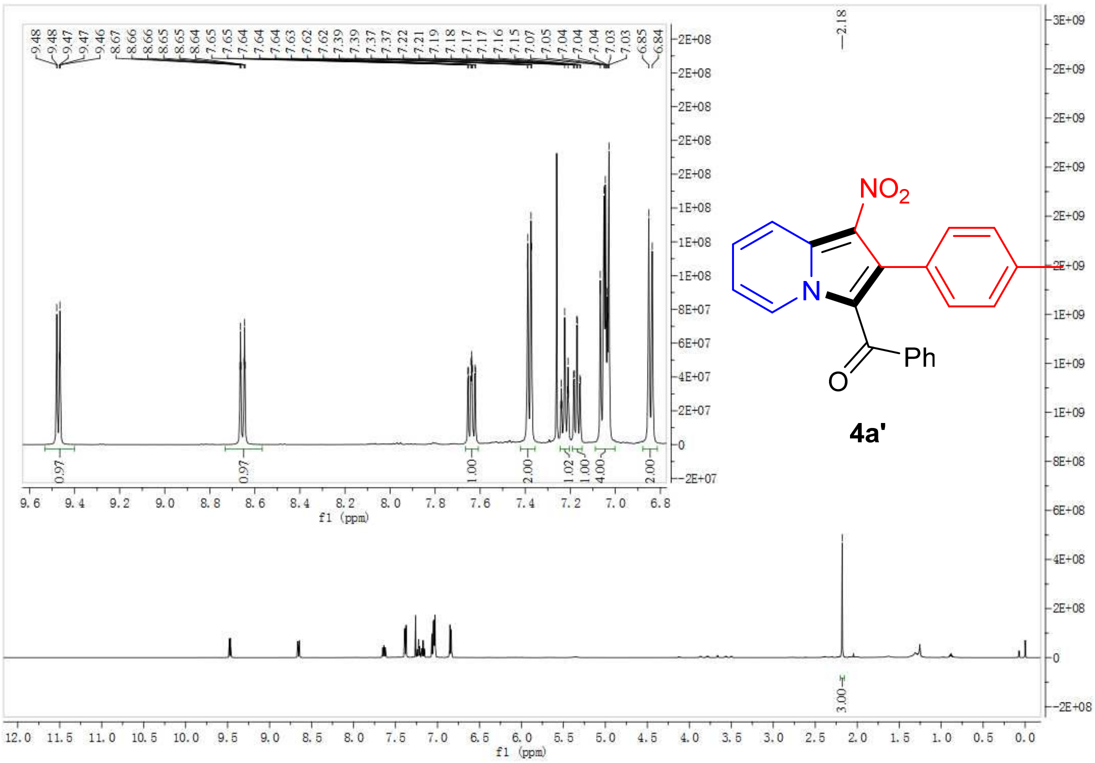

4a'

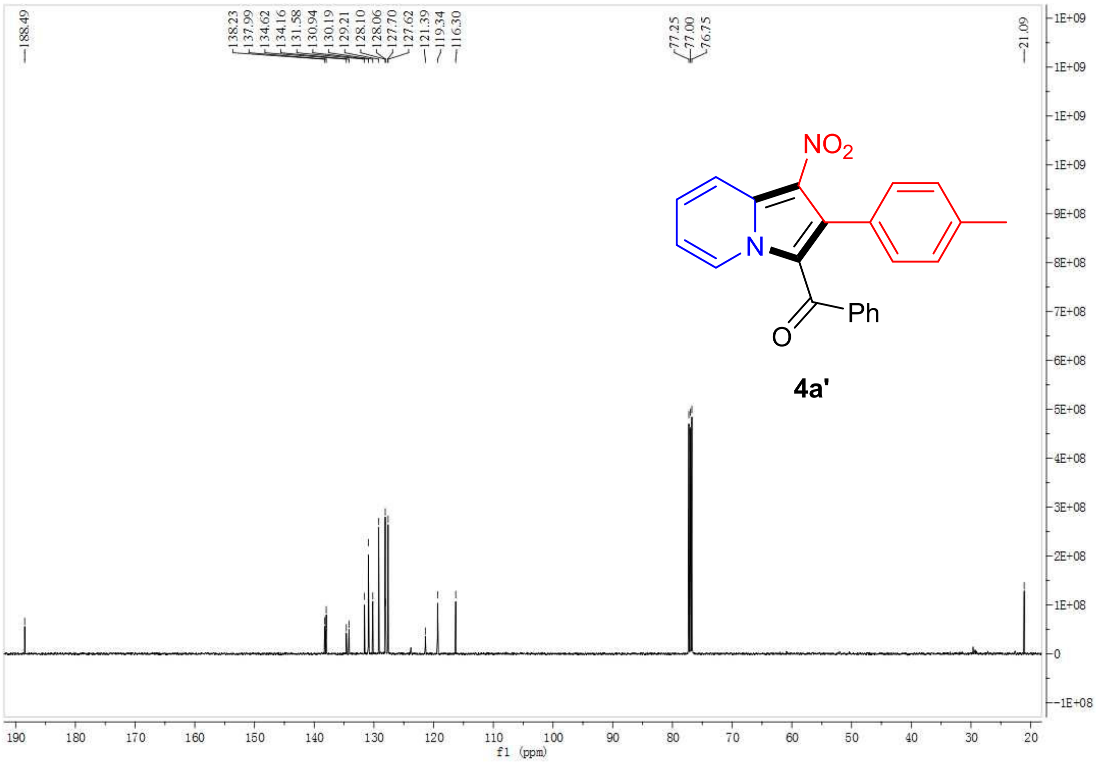

4a'

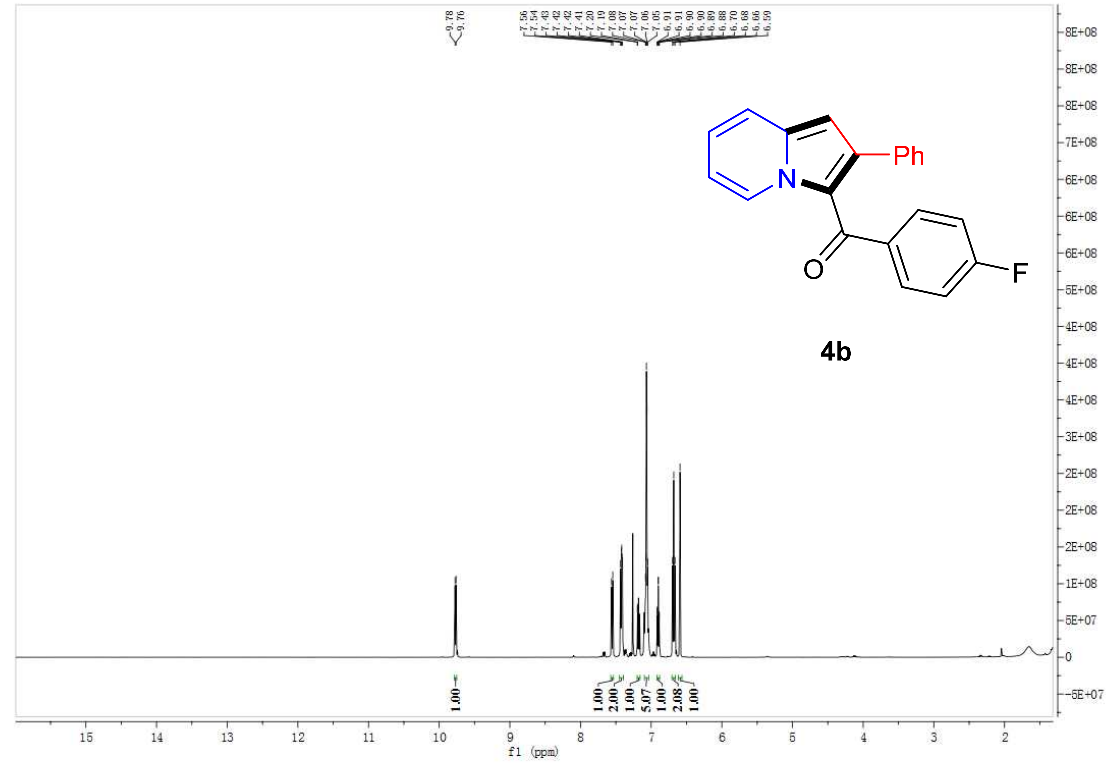

4b

^{19}F NMR (471 MHz, $CDCl_3$) δ -109.12 (s).

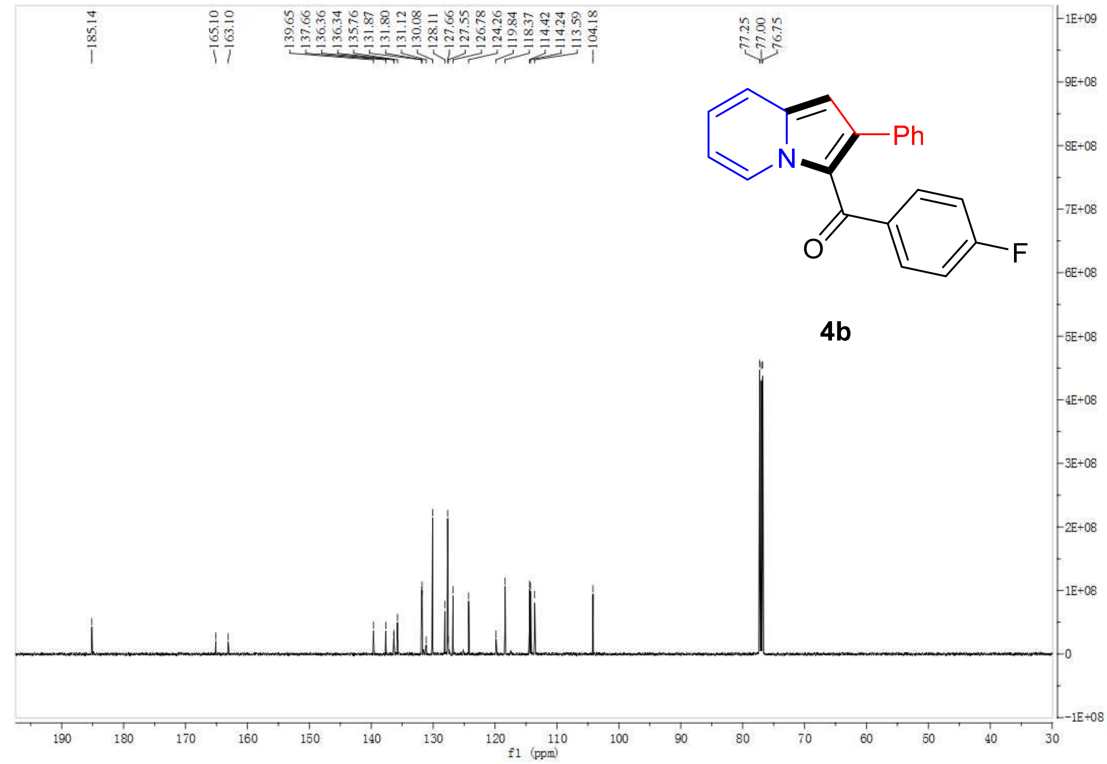

4b

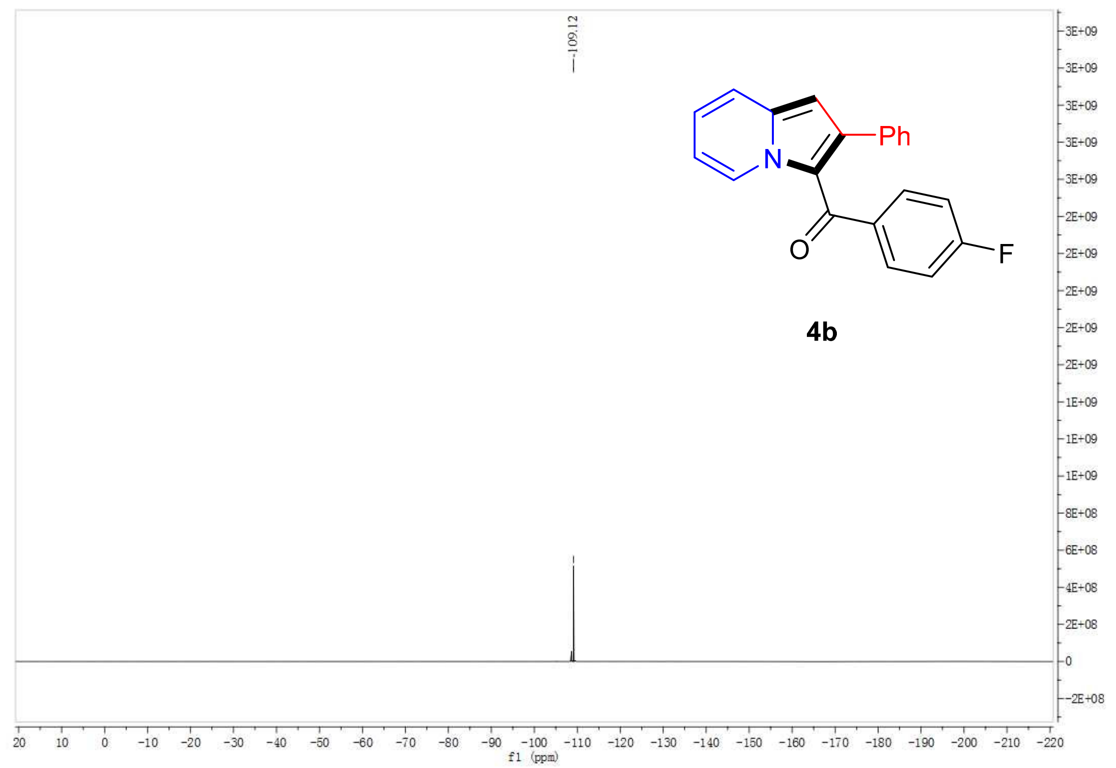

—-109.12
Ph
N
O
F
4b
3E+09
2E+09
1E+09
8E+08
6E+08
4E+08
2E+08
0
-2E+08
20 10 0 -10 -20 -30 -40 -50 -60 -70 -80 -90 -100 -110 -120 -130 -140 -150 -160 -170 -180 -190 -200 -210 -220
f1 (ppm)

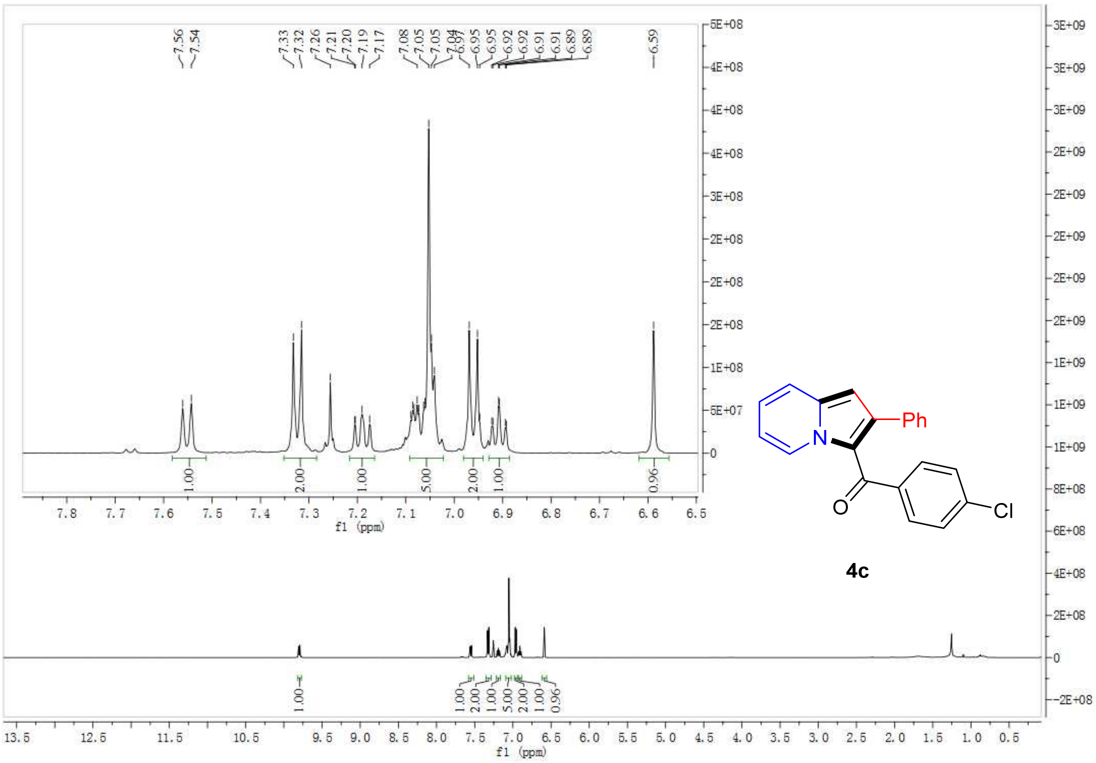

4c

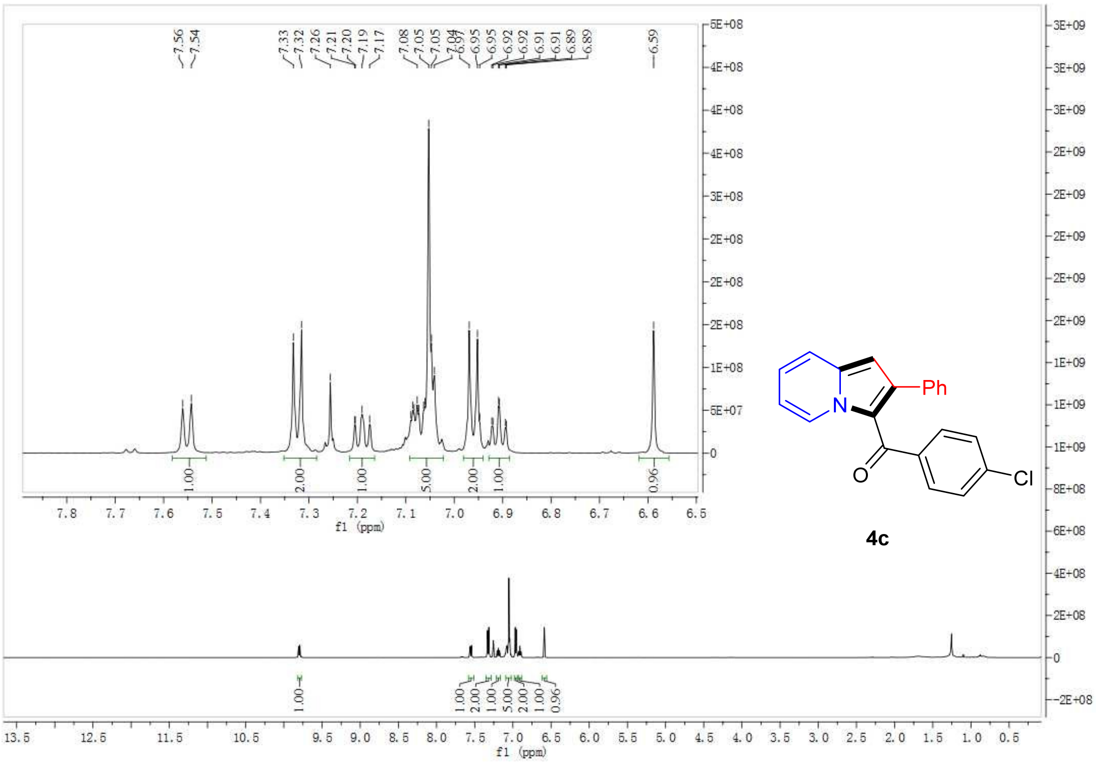
7.56
7.54
7.33
7.32
7.26
7.21
7.20
7.19
7.17
7.08
7.05
7.05
7.04
6.97
6.95
6.95
6.92
6.92
6.91
6.91
6.89
6.89
6.59
1.00
2.00
1.00
5.00
2.00
1.00
0.96
f1 (ppm)
Ph
N
O
Cl

4c

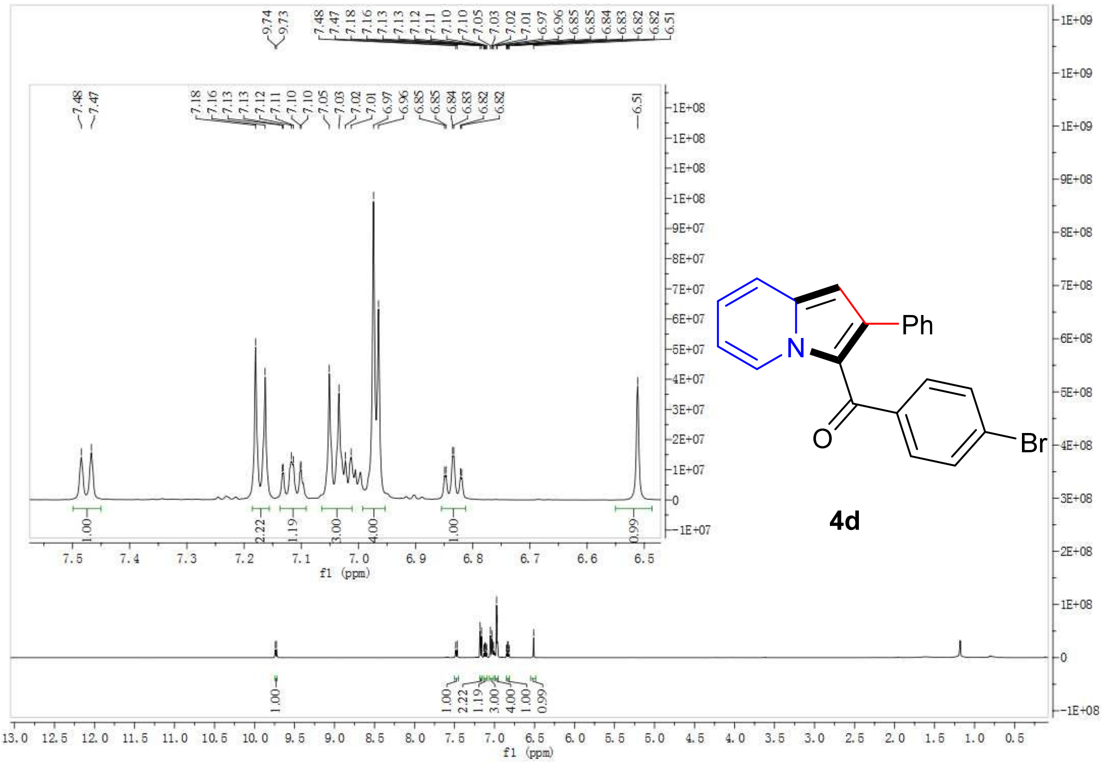
Ph
O
Br
N
4d
f1 (ppm)

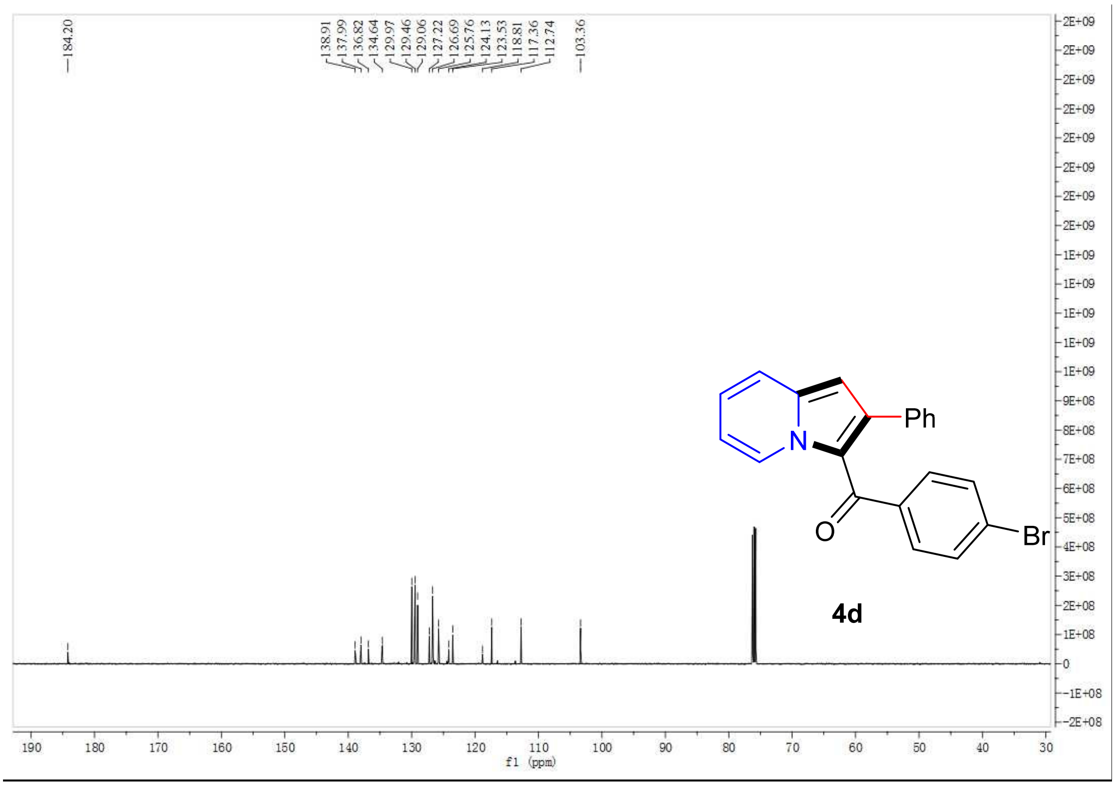
Ph
O
Br
N
4d
f1 (ppm)

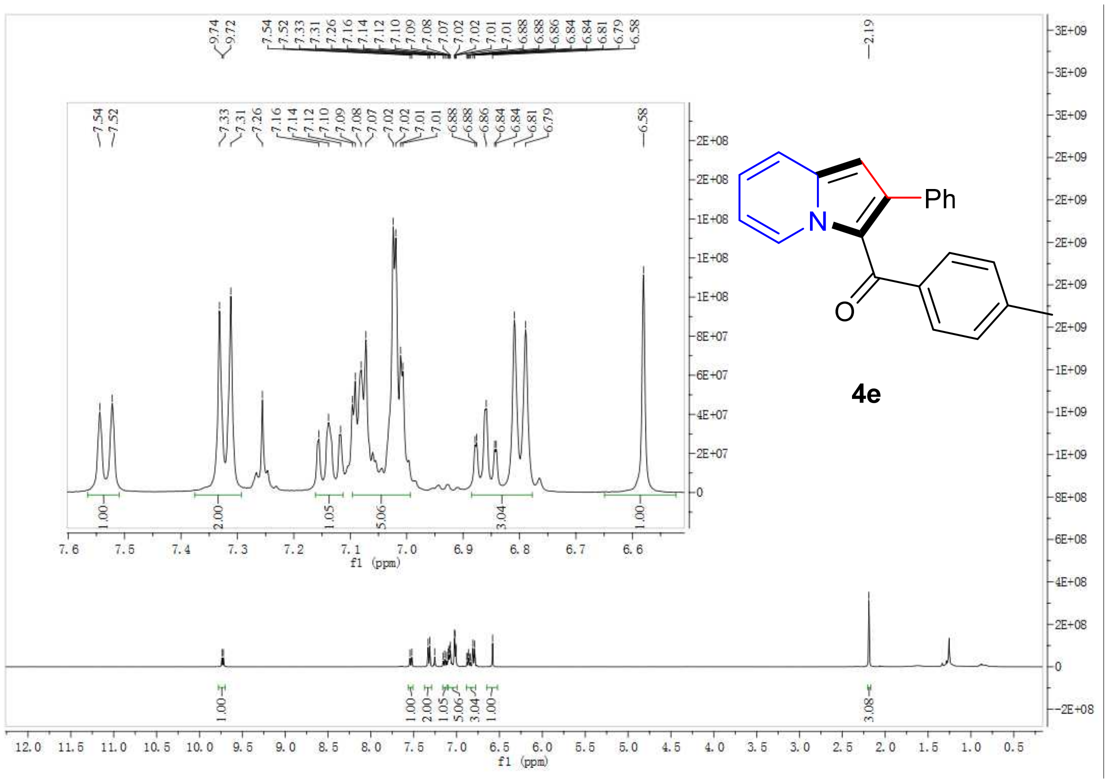
Ph
4e
f1 (ppm)

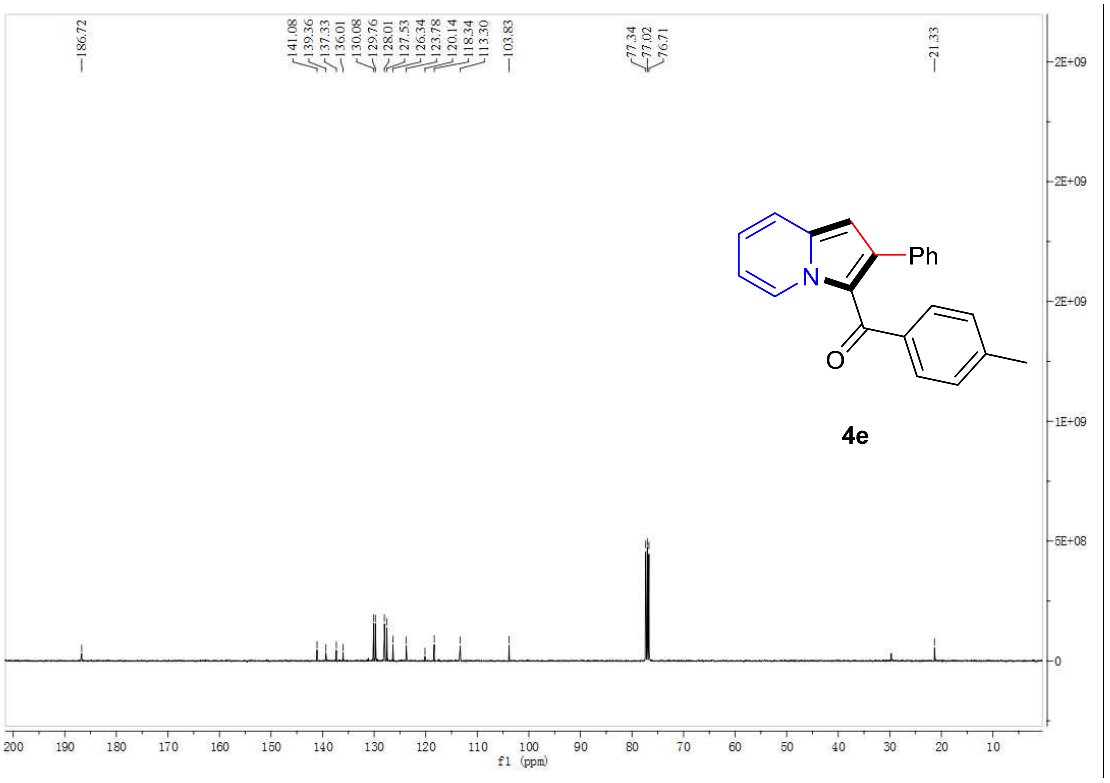
Ph
4e
f1 (ppm)

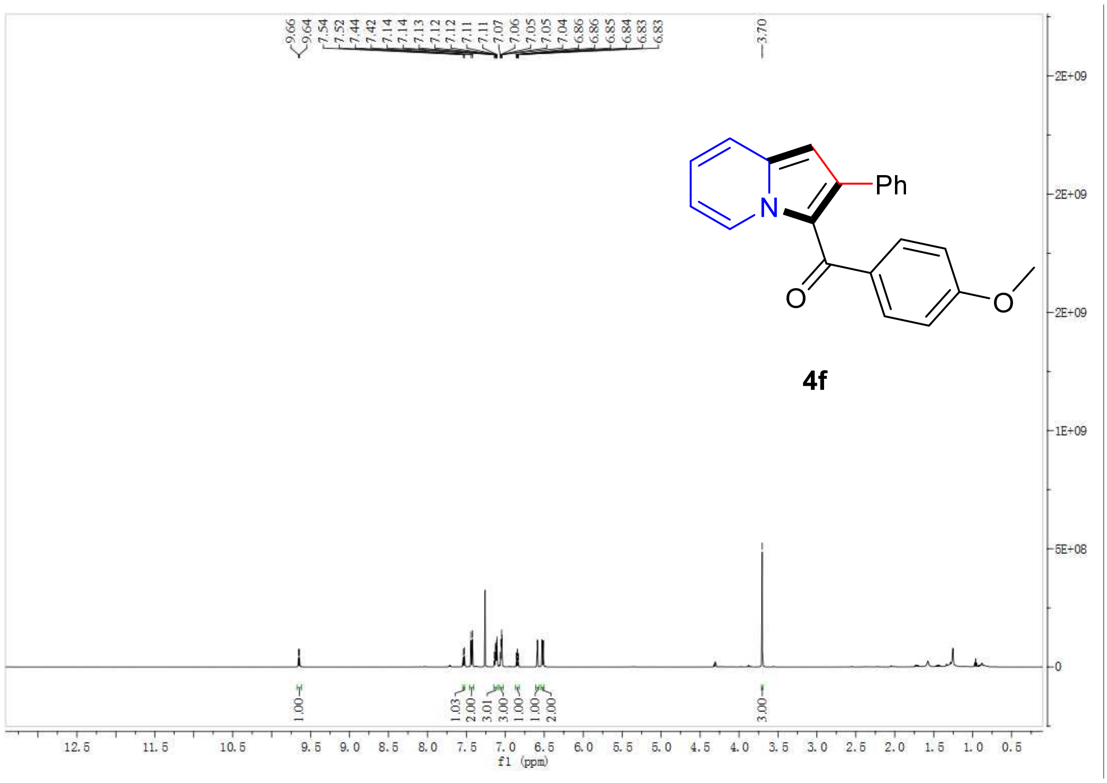

9.66
9.64
7.54
7.52
7.44
7.42
7.14
7.14
7.13
7.12
7.12
7.11
7.11
7.07
7.06
7.05
7.05
7.04
6.86
6.86
6.85
6.84
6.83
6.83
3.70
Ph
N
O
O
4f
1.00
1.03
2.00
3.01
3.00
1.00
1.00
2.00
3.00
f1 (ppm)

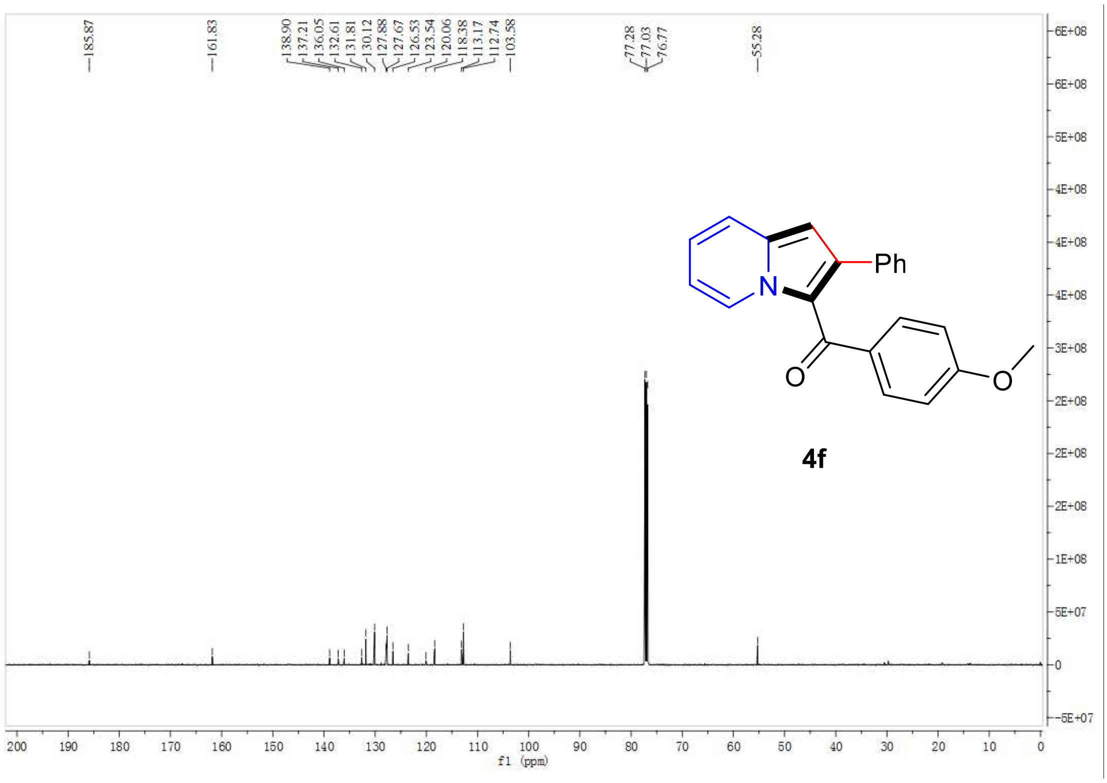

185.87
161.83
138.90
137.21
136.05
132.61
131.81
130.12
127.88
127.67
126.53
123.54
120.06
118.38
113.17
112.74
103.58
77.28
77.03
76.77
55.28
Ph
N
O
O
4f
f1 (ppm)

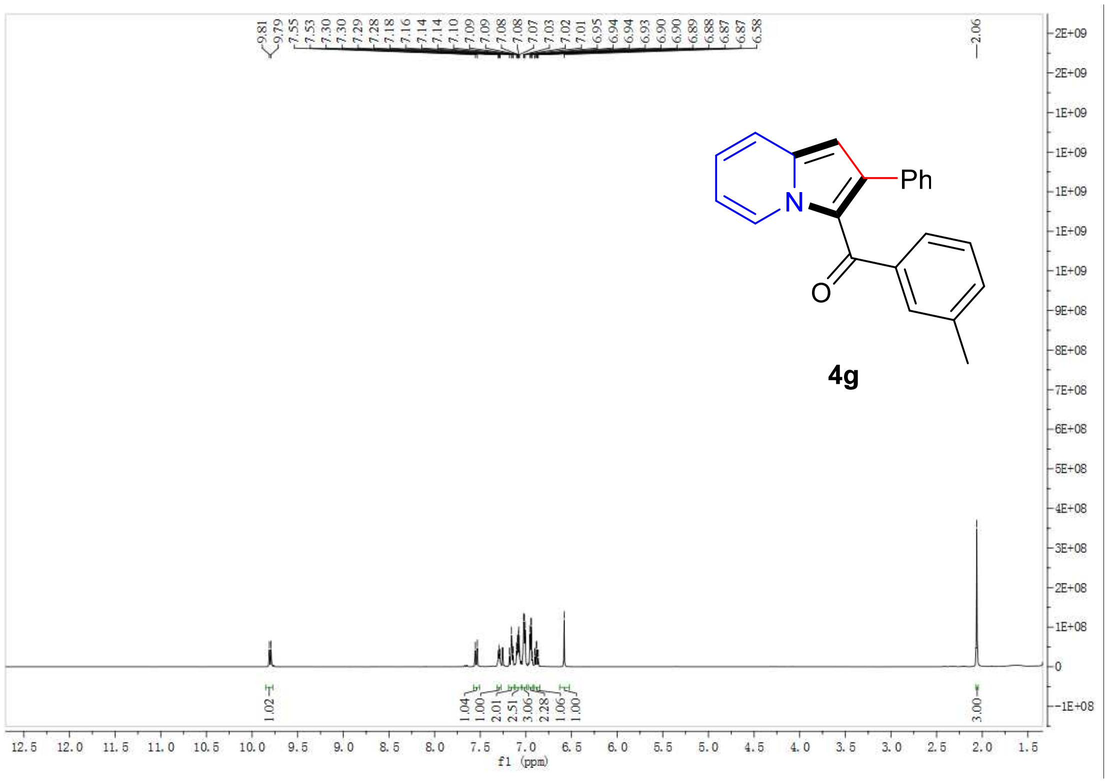
4g

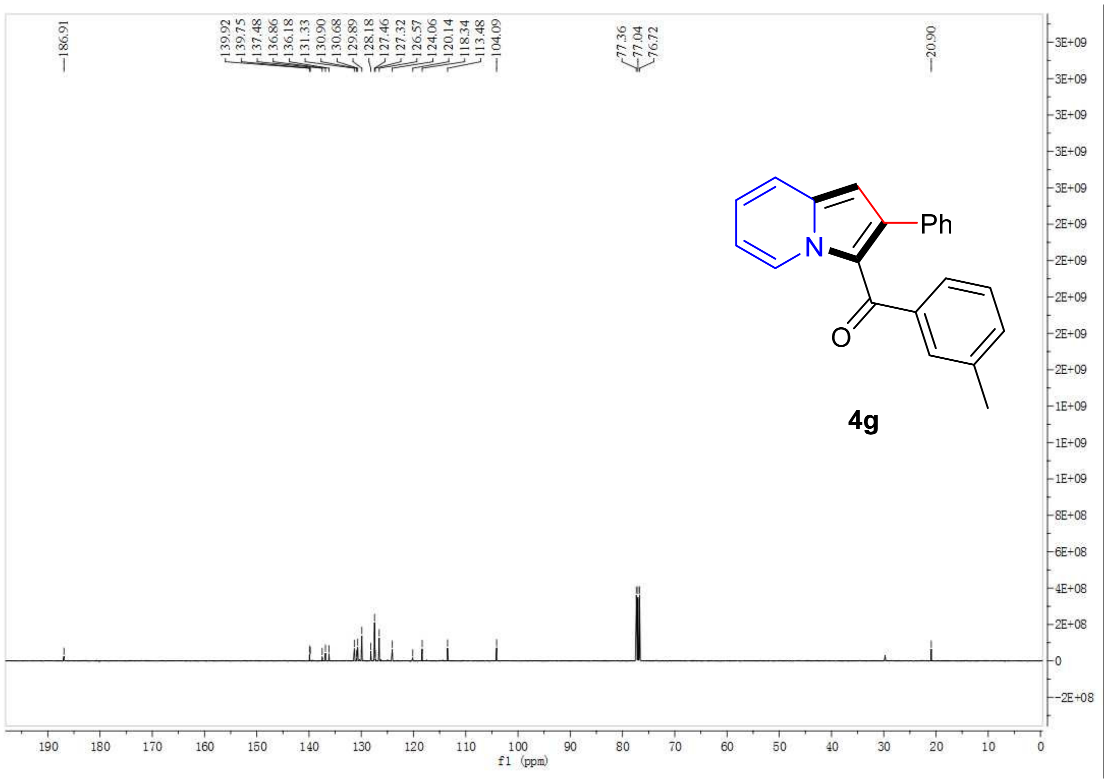
4g

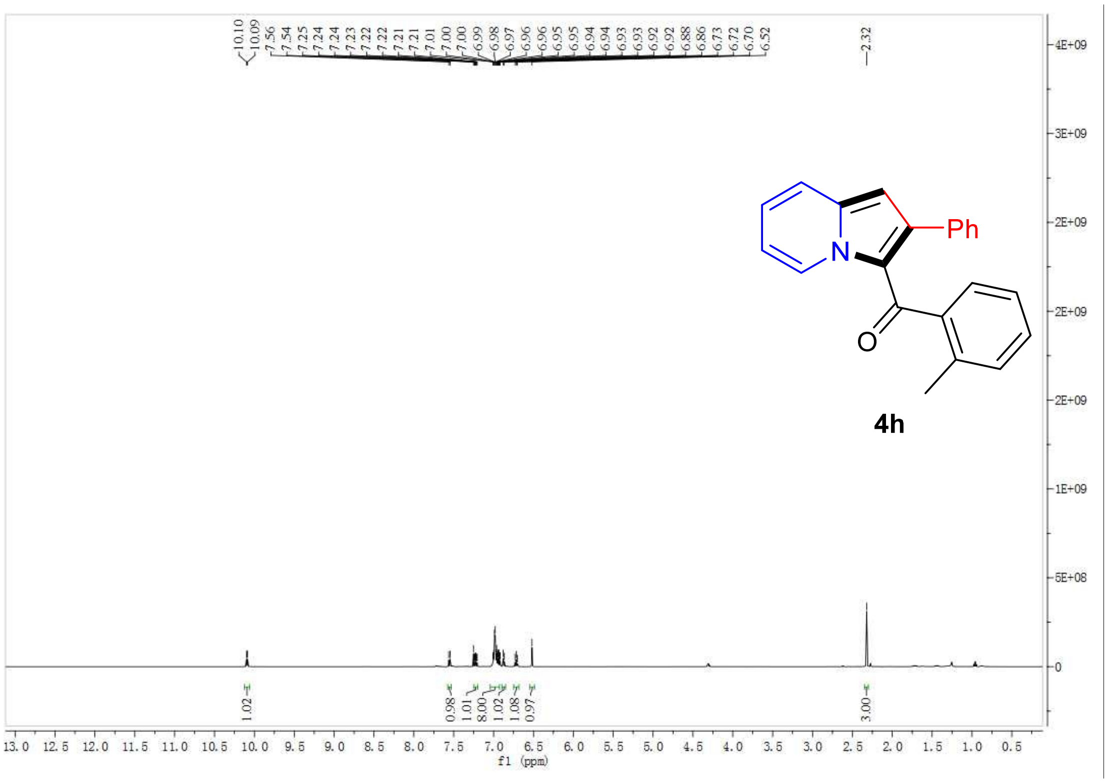
10.10
10.09
7.56
7.54
7.25
7.24
7.24
7.23
7.22
7.22
7.21
7.21
7.01
7.00
7.00
6.99
6.98
6.97
6.96
6.96
6.95
6.95
6.94
6.94
6.93
6.93
6.92
6.92
6.88
6.86
6.73
6.72
6.70
6.52
2.32
N
Ph
O
4h
1.02
0.98
1.01
8.00
1.02
1.08
0.97
3.00
f1 (ppm)

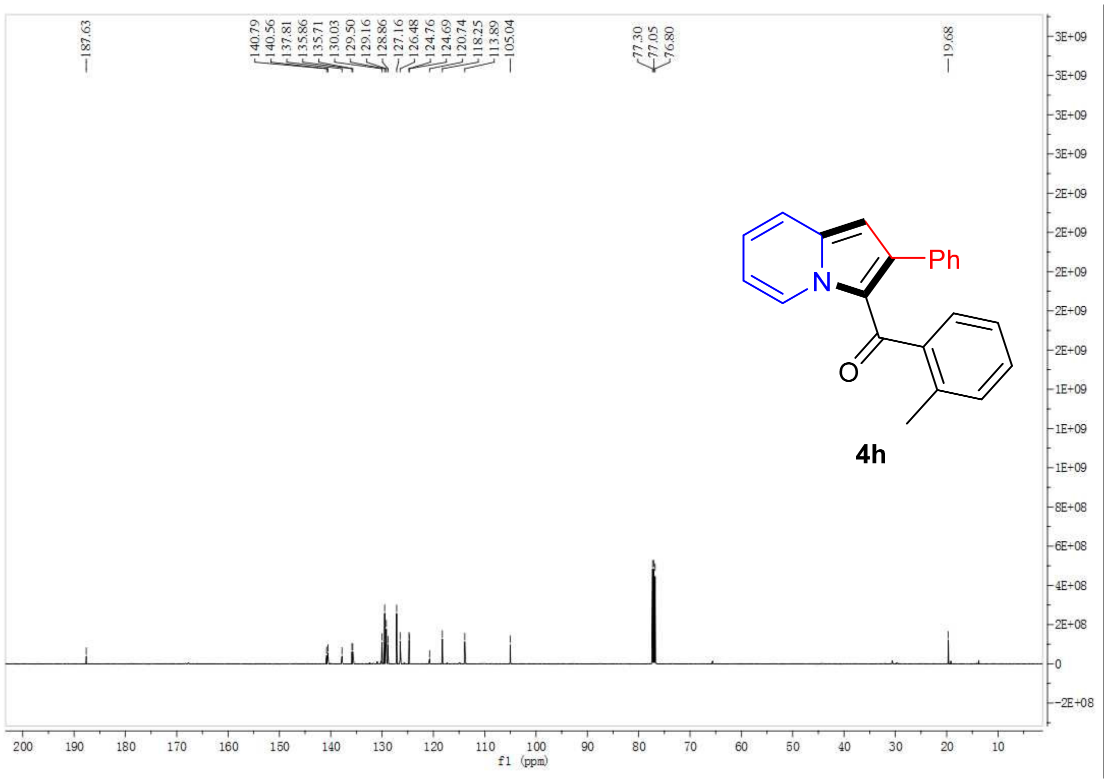
187.63
140.79
140.56
137.81
135.86
135.71
130.03
129.50
129.16
128.86
127.16
126.48
124.76
124.69
120.74
118.25
113.89
105.04
77.30
77.05
76.80
19.68
N
Ph
O
4h
f1 (ppm)

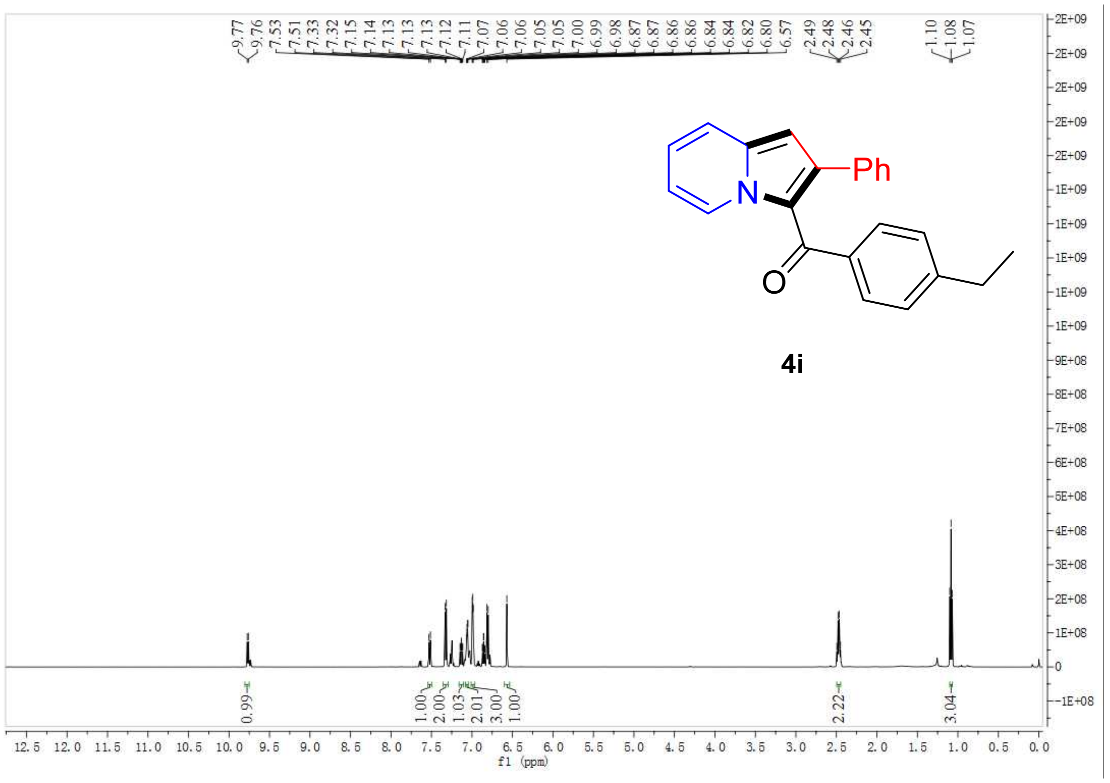

4i
f1 (ppm)

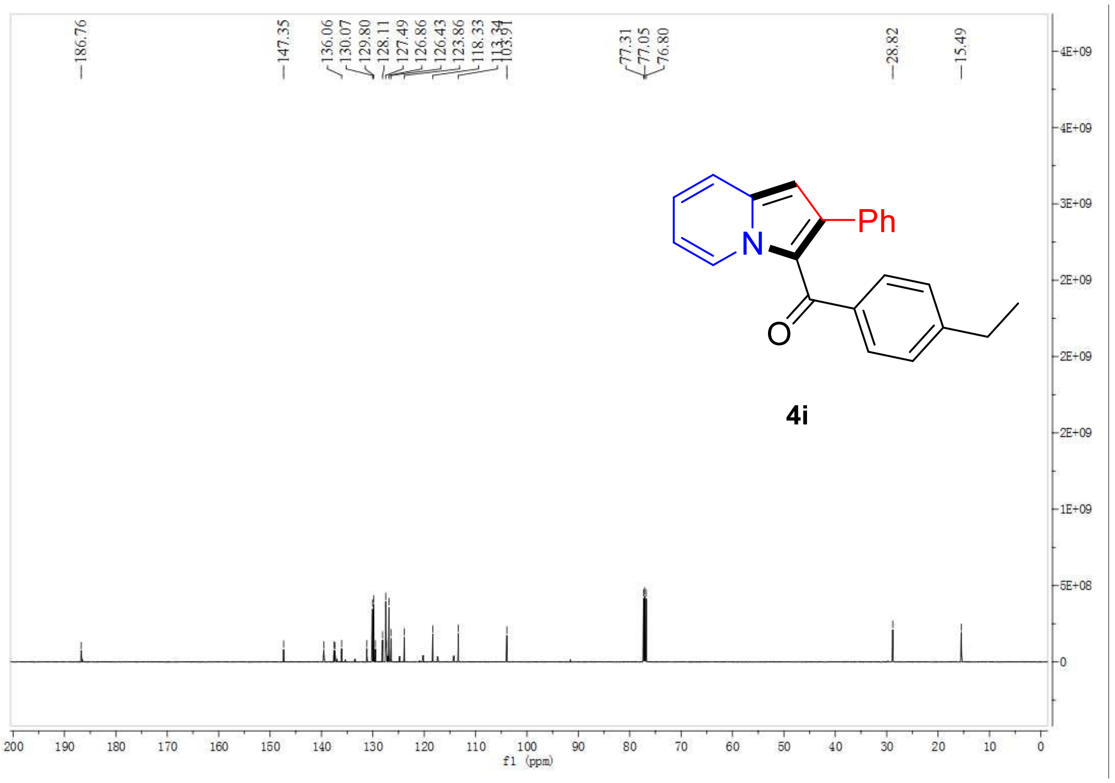

4i
f1 (ppm)

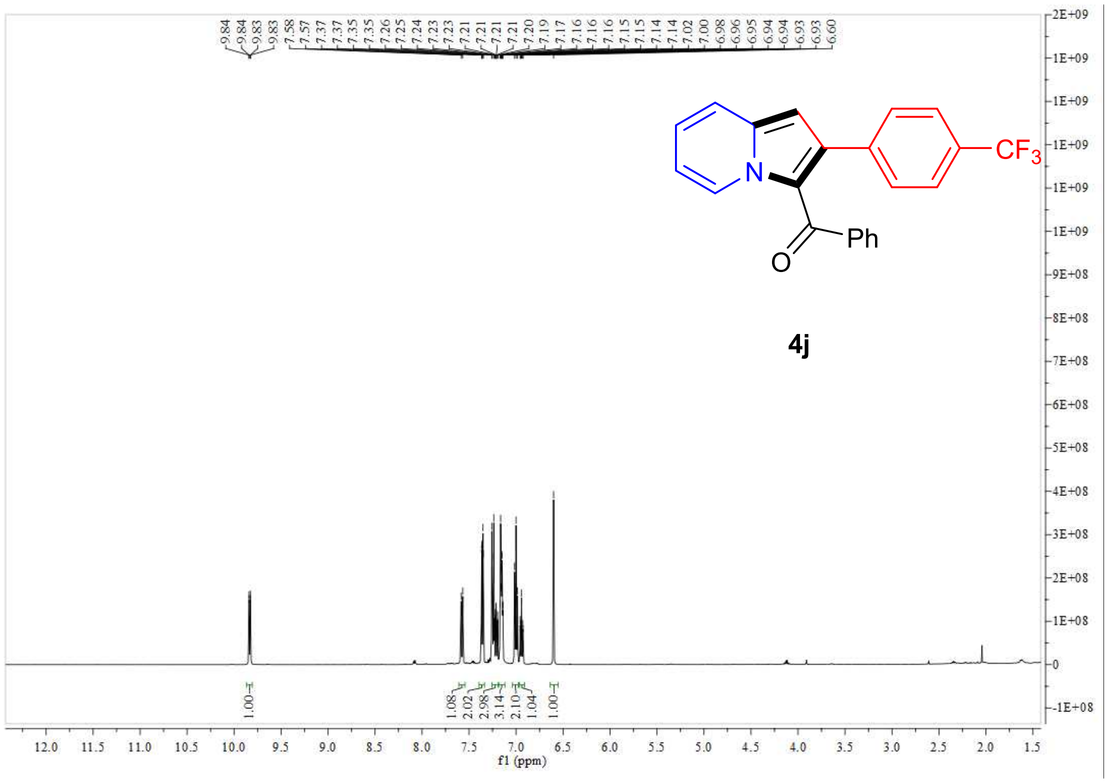

4j
f1 (ppm)

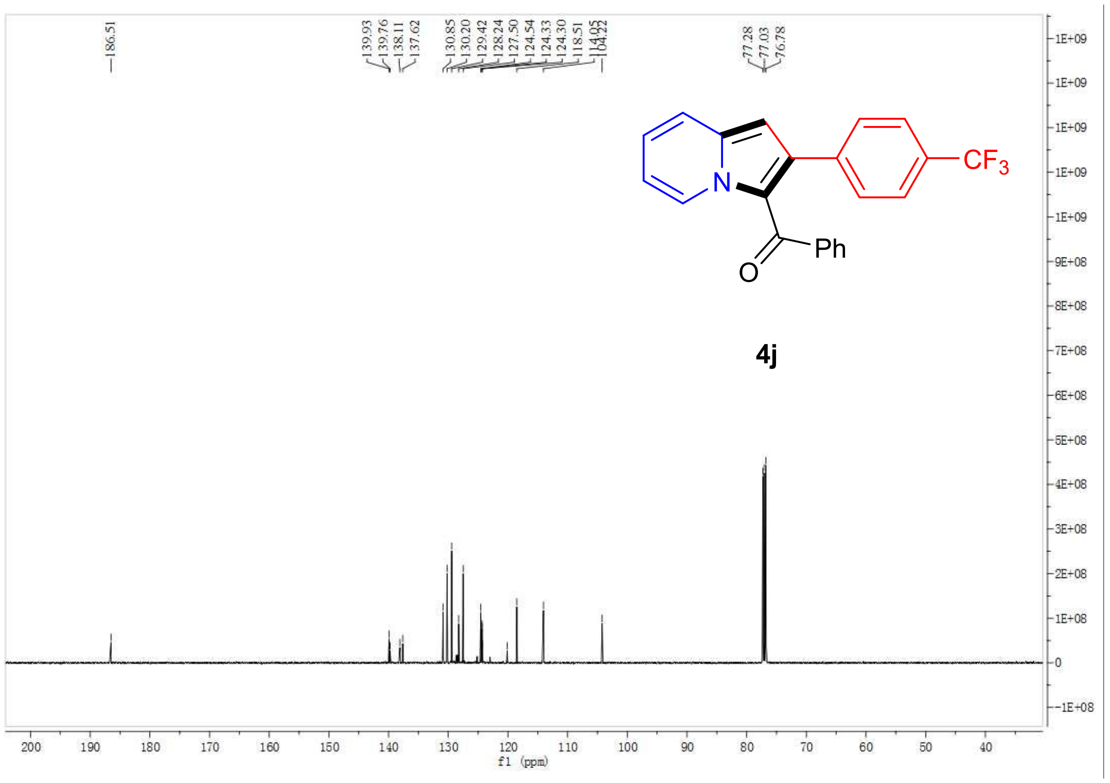

4j
f1 (ppm)

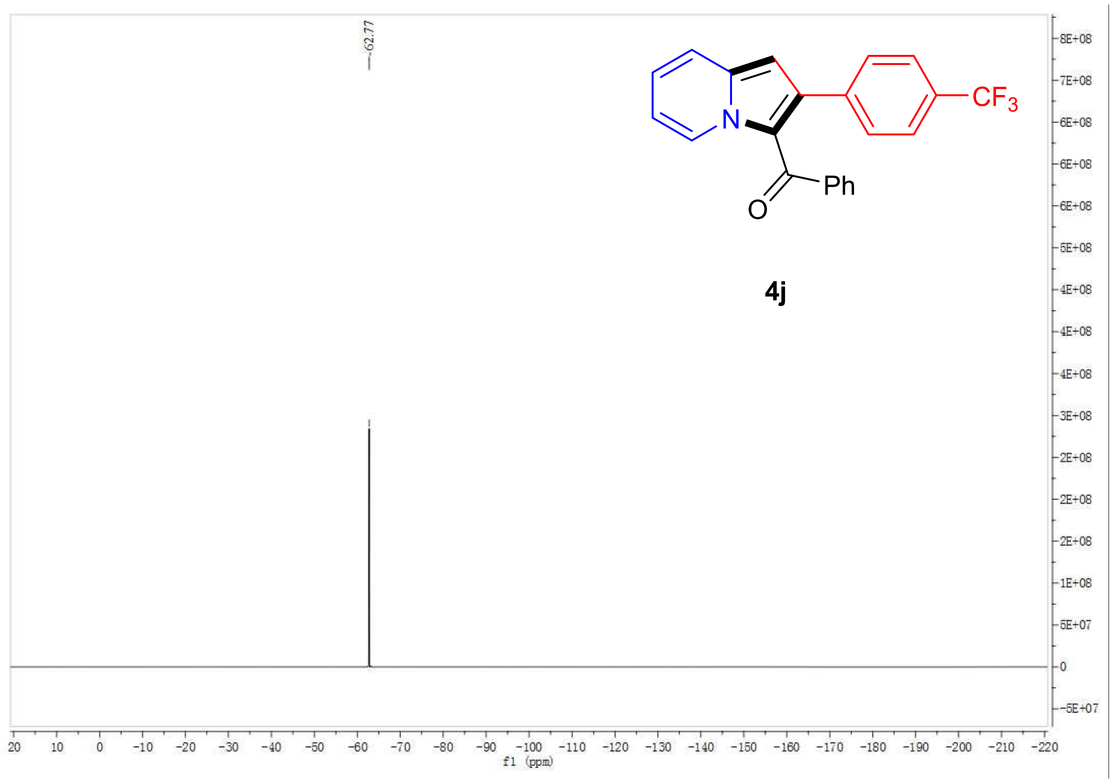

-62.77
CF3
N
Ph
O
4j
f1 (ppm)

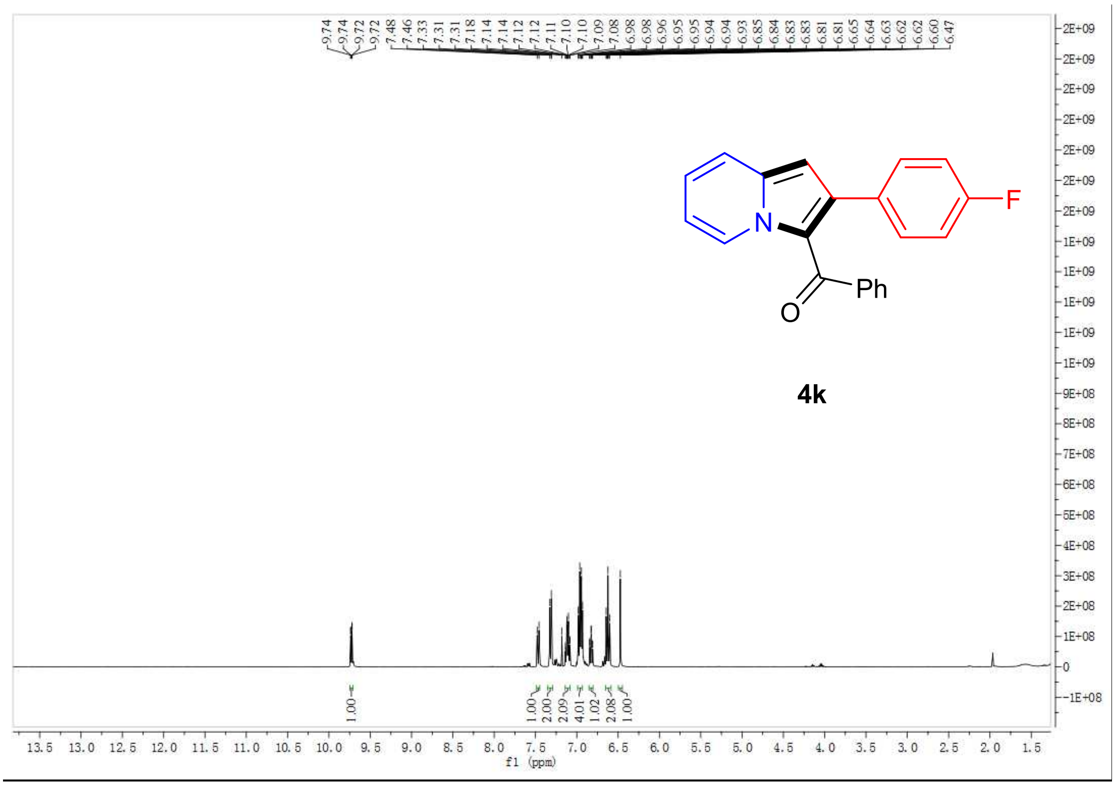

N
F
Ph
O
4k
f1 (ppm)

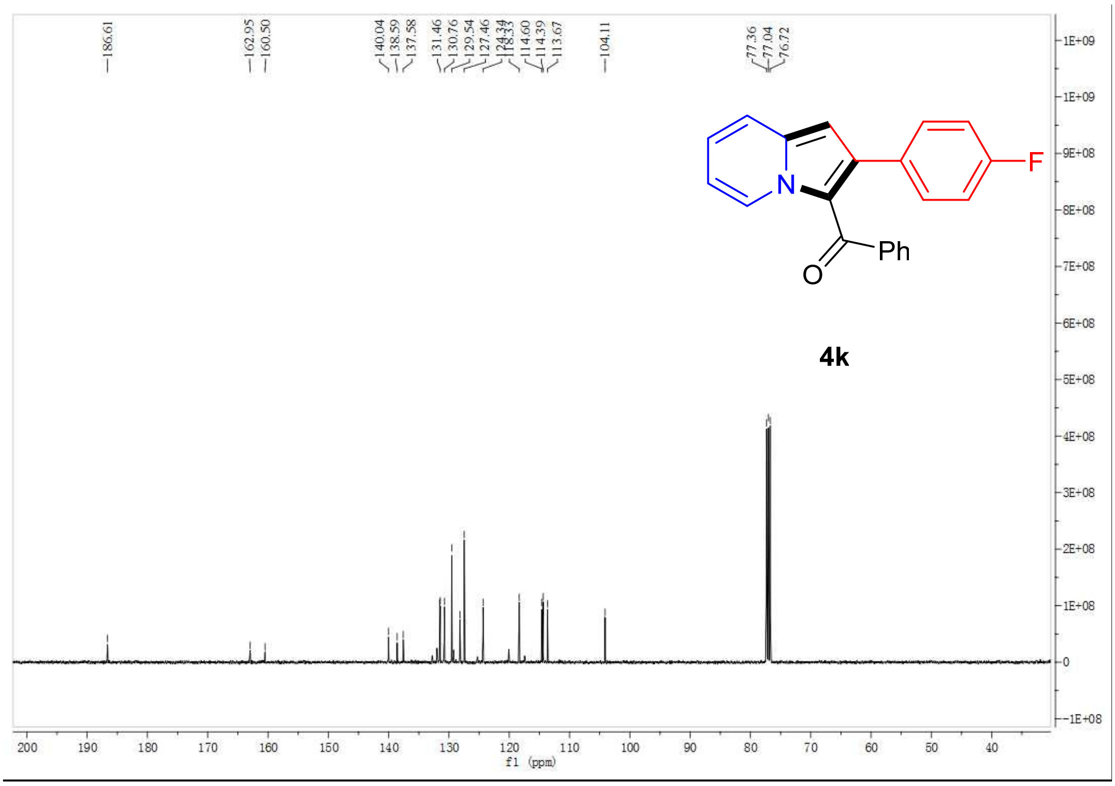
186.61
162.95
160.50
140.04
138.59
137.58
131.46
130.76
129.54
127.46
124.34
118.33
114.60
114.39
113.67
104.11
77.36
77.04
76.72
N
F
Ph
O
4k
f1 (ppm)

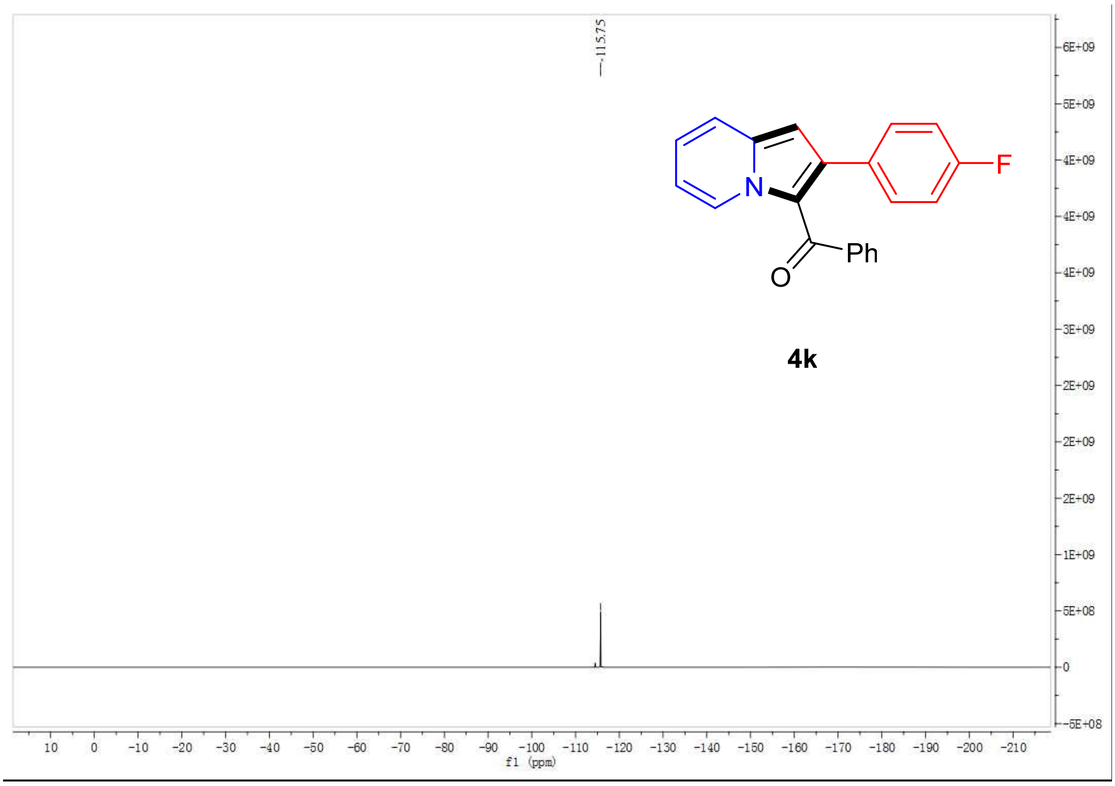
-115.75
N
F
Ph
O
4k
f1 (ppm)

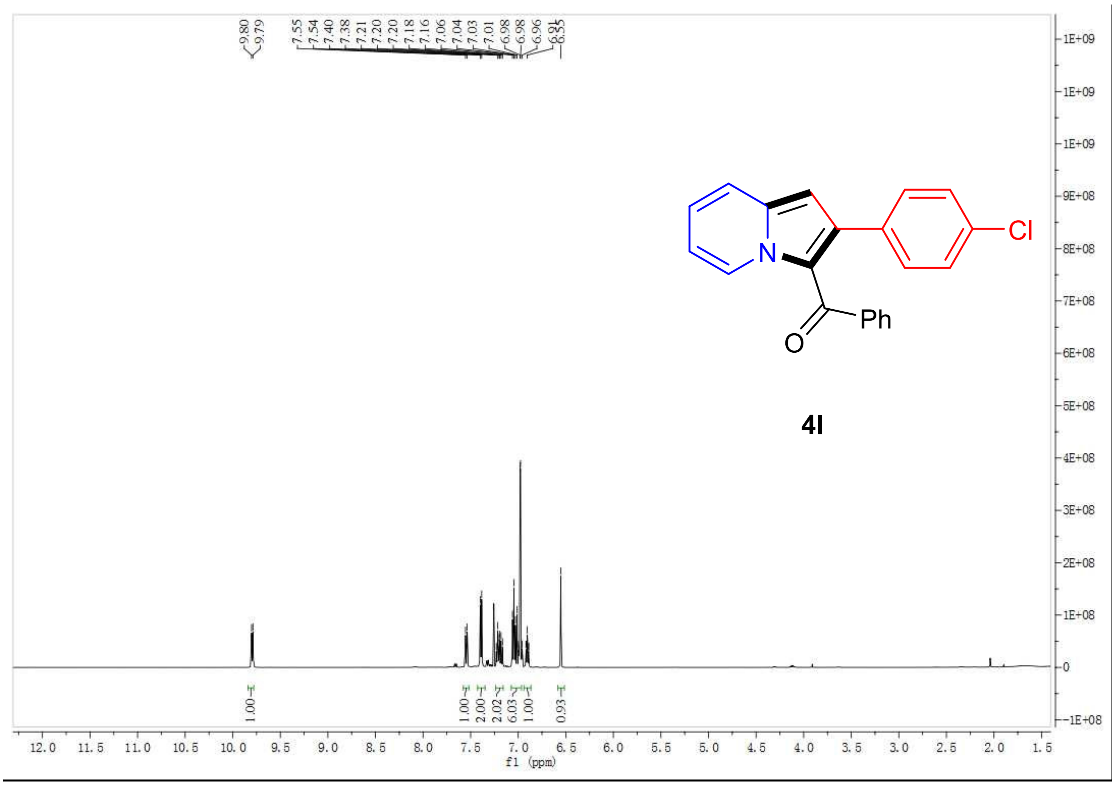

Cl
Ph
O
N
4l
f1 (ppm)

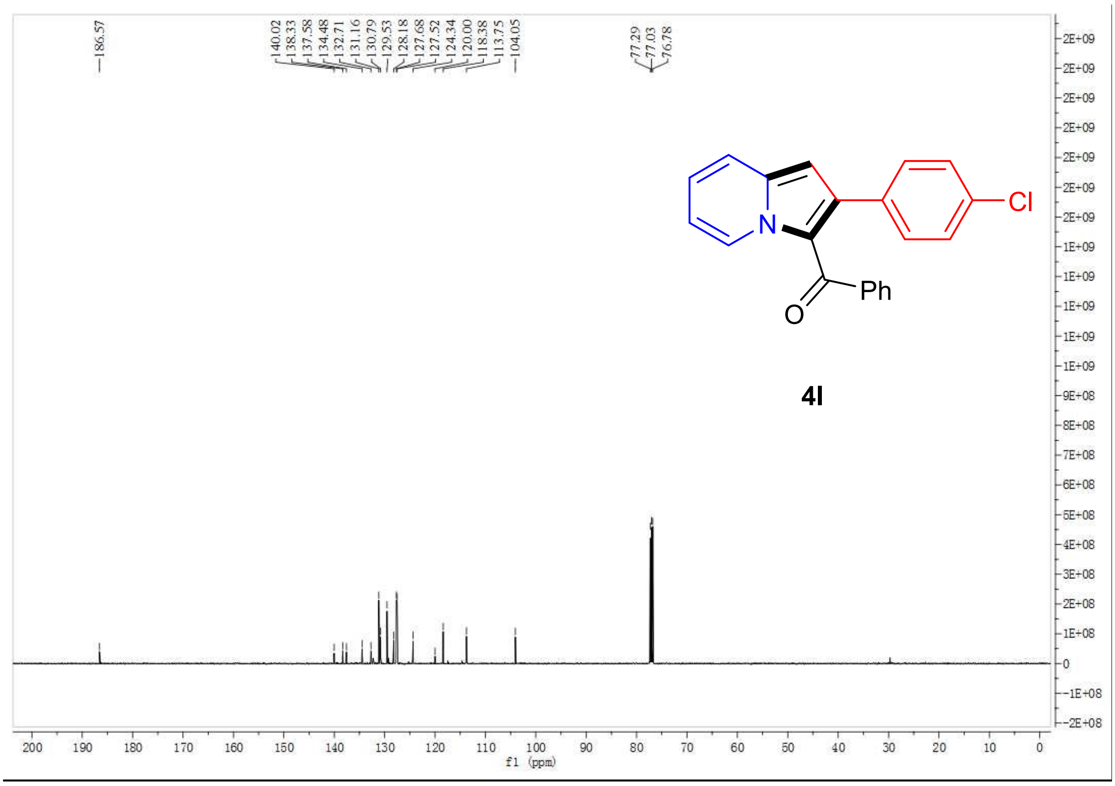

Cl
Ph
O
N
4l
f1 (ppm)

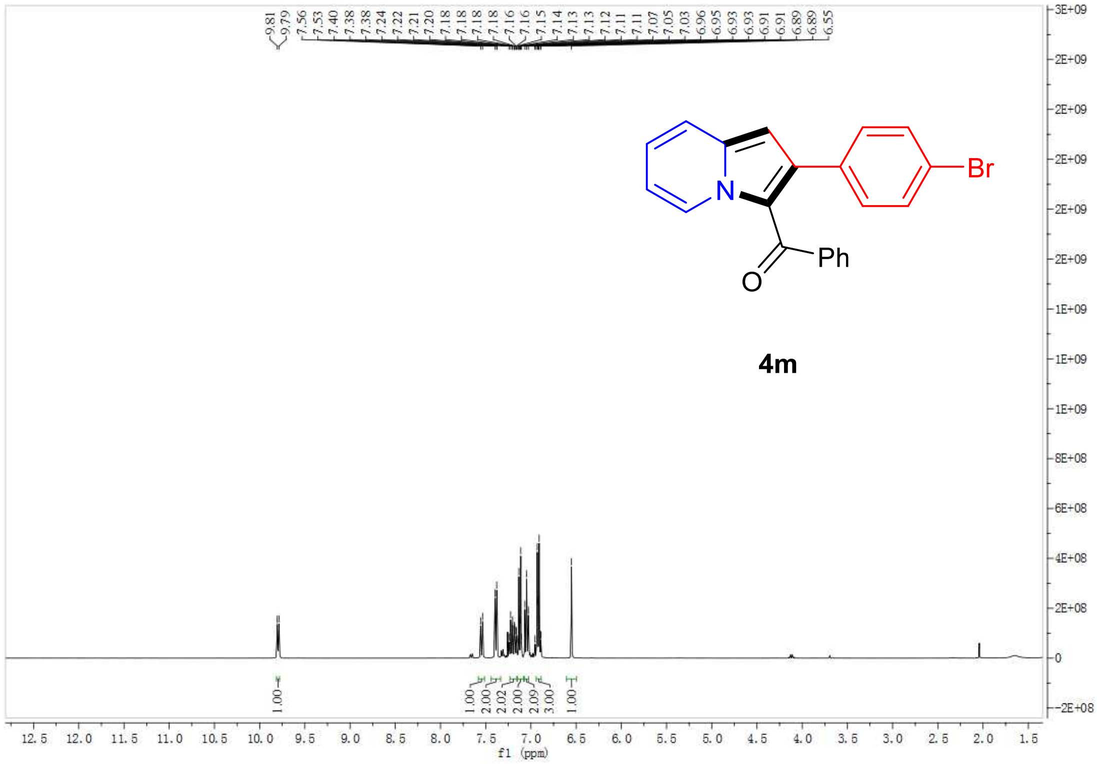
4m
f1 (ppm)

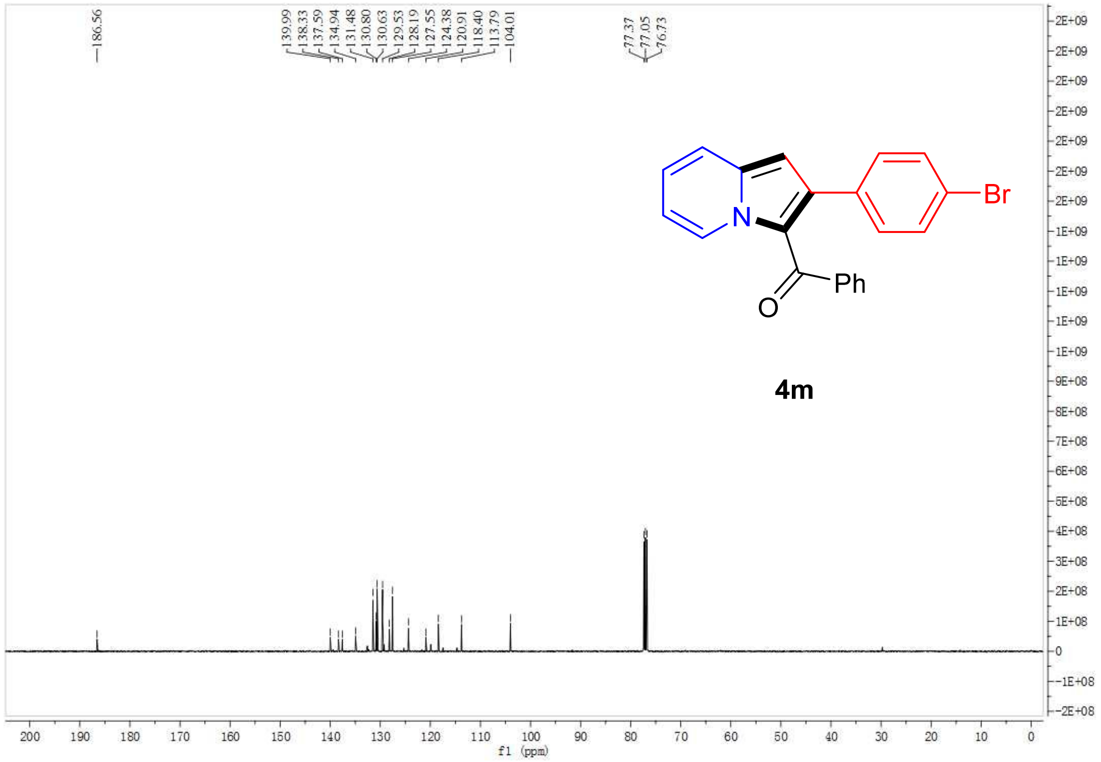
4m
f1 (ppm)

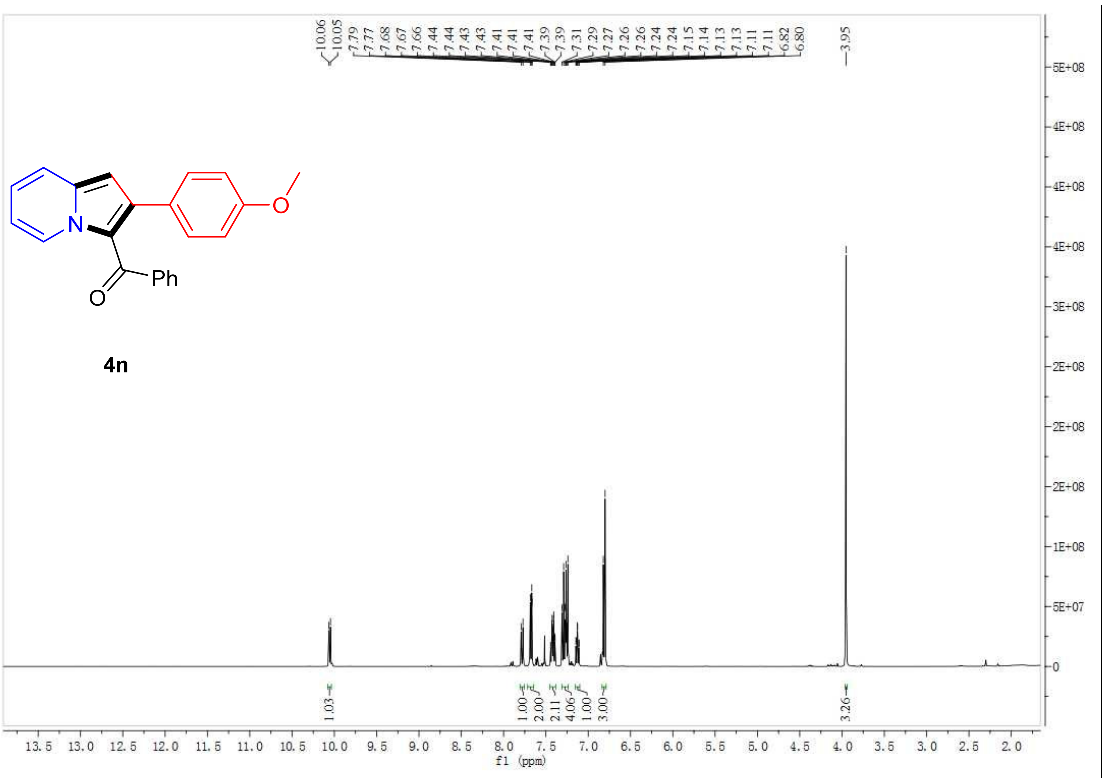
10.06
10.05
7.79
7.77
7.68
7.67
7.66
7.44
7.44
7.43
7.43
7.41
7.41
7.41
7.39
7.39
7.31
7.29
7.27
7.26
7.26
7.24
7.24
7.15
7.14
7.13
7.13
7.11
7.11
6.82
6.80
3.95
N
O
Ph
4n
1.03
1.00
2.00
2.11
4.06
1.00
3.00
3.26
f1 (ppm)

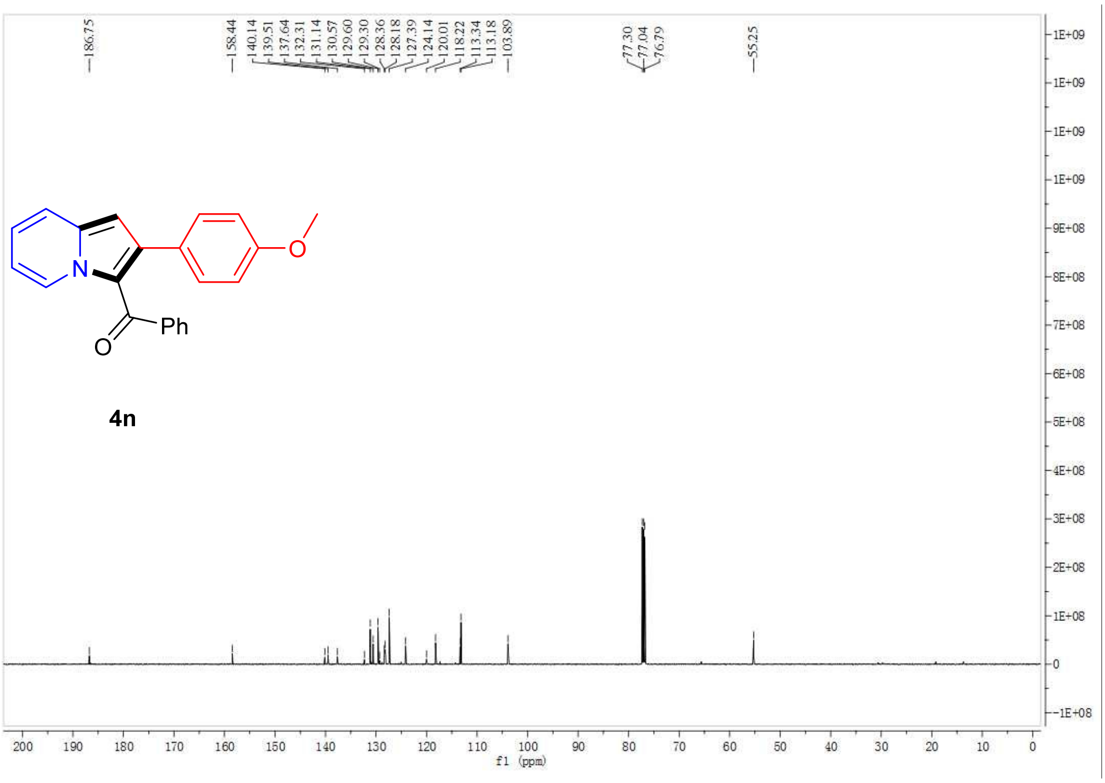
186.75
158.44
140.14
139.51
137.64
132.31
131.14
130.57
129.60
129.30
128.36
128.18
127.39
124.14
120.01
118.22
113.34
113.18
103.89
77.30
77.04
76.79
55.25
N
O
Ph
4n
f1 (ppm)

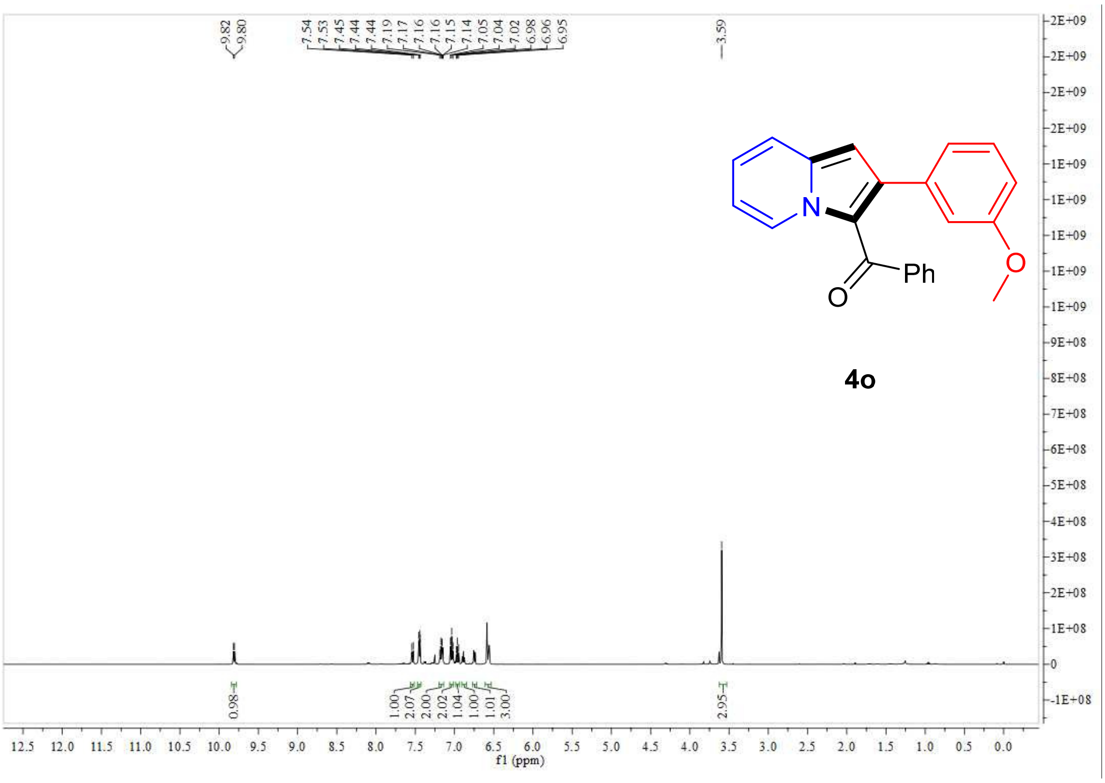
9.82
9.80
7.54
7.53
7.45
7.44
7.44
7.19
7.17
7.16
7.16
7.15
7.14
7.05
7.04
7.02
6.98
6.96
6.95
3.59
N
O
Ph
4o
0.98
1.00
2.07
2.00
2.02
1.04
1.00
1.01
3.00
2.95
f1 (ppm)

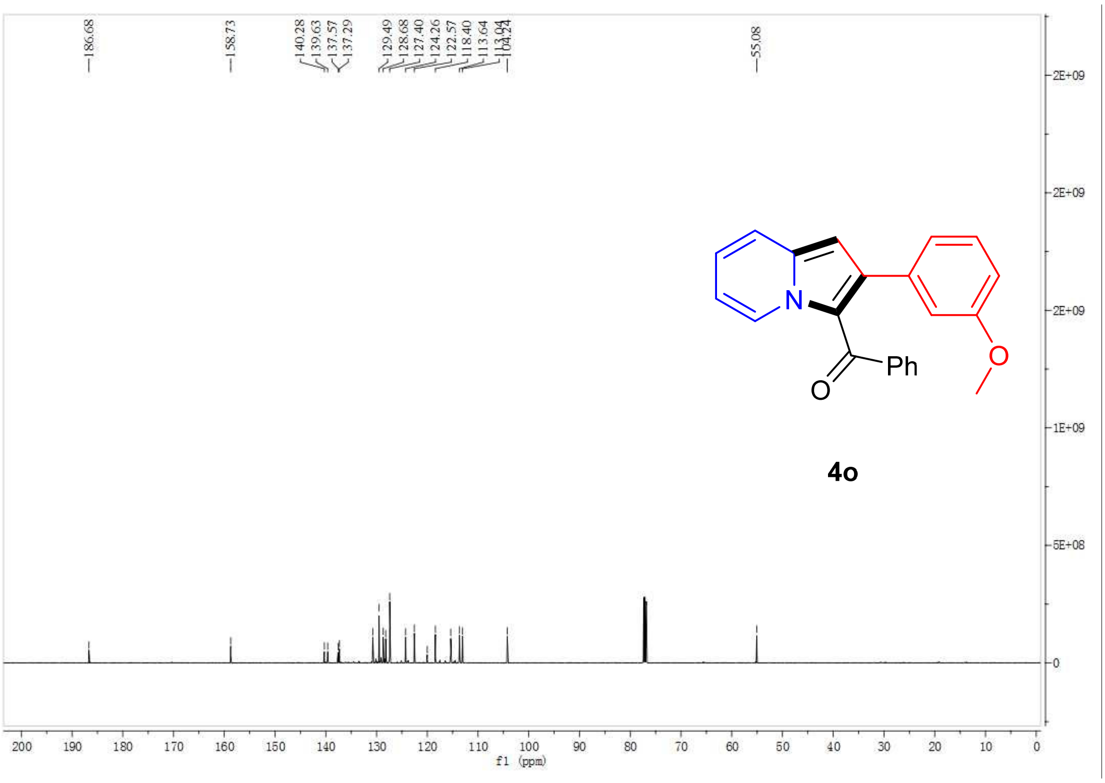
186.68
158.73
140.28
139.63
137.57
137.29
129.49
128.68
127.40
124.26
122.57
118.40
113.64
113.04
104.24
55.08
N
O
Ph
4o
f1 (ppm)

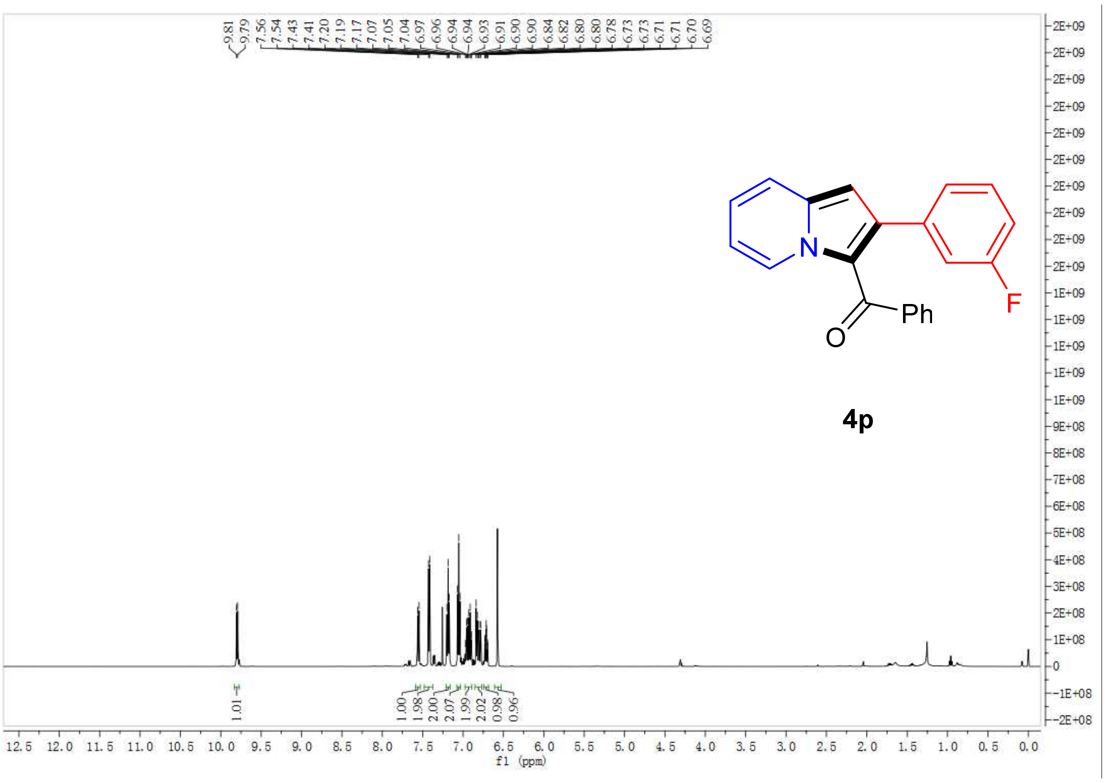

9.81
9.79
7.56
7.54
7.43
7.41
7.20
7.19
7.17
7.07
7.05
7.04
6.97
6.96
6.94
6.94
6.93
6.91
6.90
6.90
6.84
6.82
6.80
6.80
6.78
6.73
6.73
6.71
6.71
6.70
6.69
N
Ph
O
F
4p
1.01
1.00
1.98
2.00
2.07
1.99
2.02
0.98
0.96
f1 (ppm)

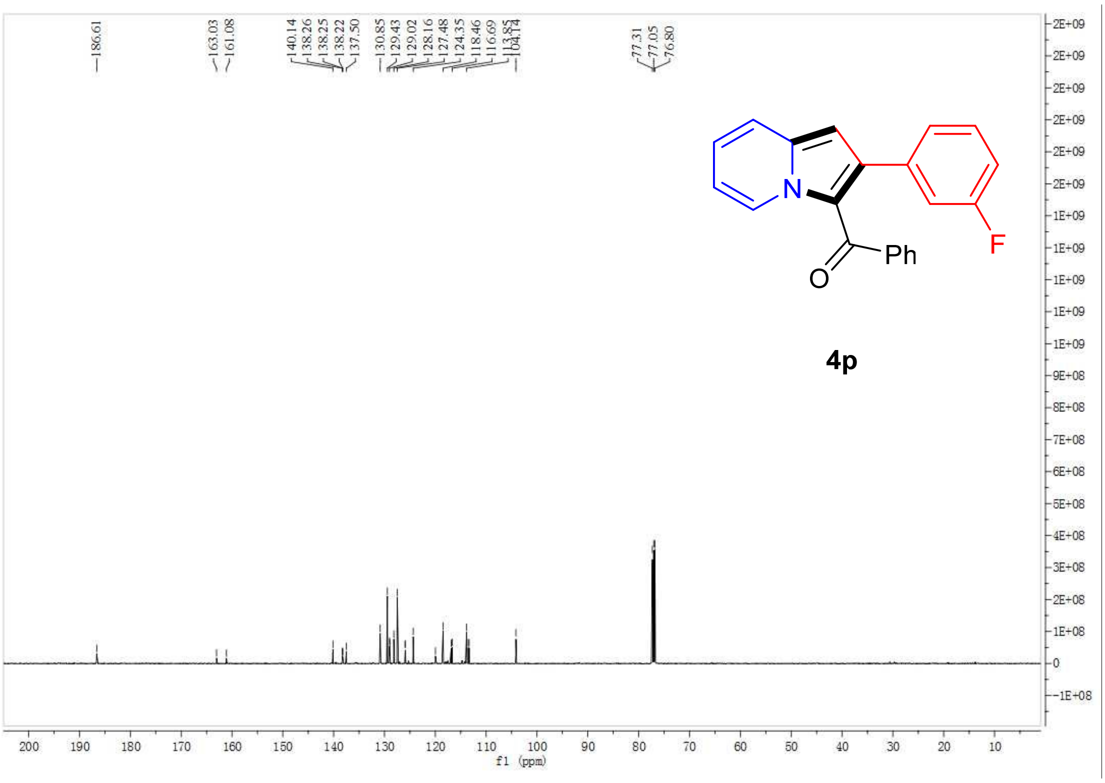

186.61
163.03
161.08
140.14
138.26
138.25
138.22
137.50
130.85
129.43
129.02
128.16
127.48
124.35
118.46
116.69
113.85
104.14
77.31
77.05
76.80
N
Ph
O
F
4p
f1 (ppm)

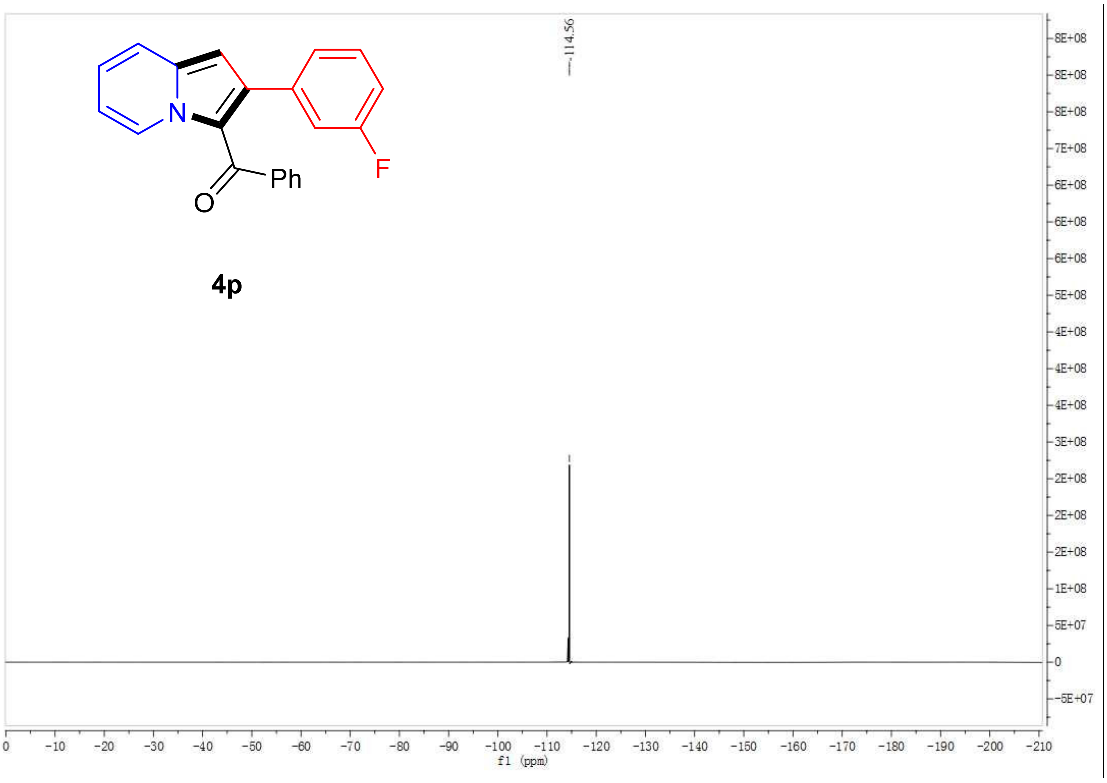
N
Ph
O
F
4p
-114.56
8E+08
8E+08
8E+08
7E+08
6E+08
6E+08
6E+08
5E+08
4E+08
4E+08
4E+08
3E+08
2E+08
2E+08
2E+08
1E+08
5E+07
0
-5E+07
0
-10
-20
-30
-40
-50
-60
-70
-80
-90
-100
-110
-120
-130
-140
-150
-160
-170
-180
-190
-200
-210
f1 (ppm)

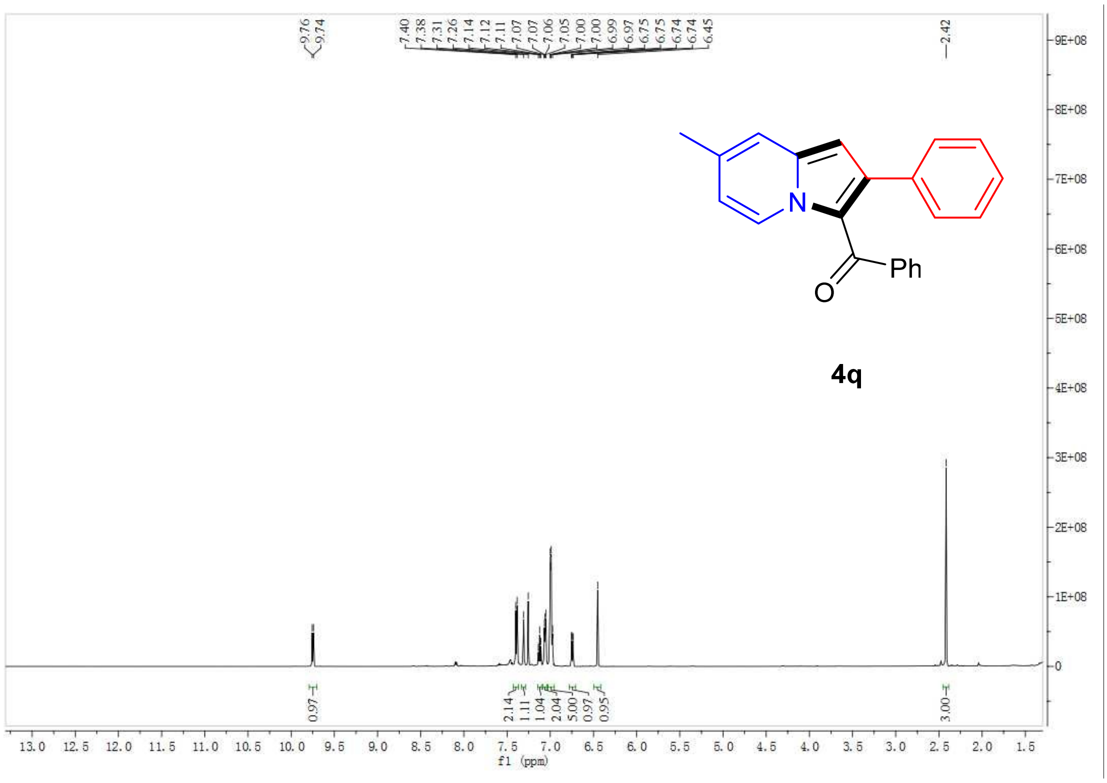
4q
f1 (ppm)

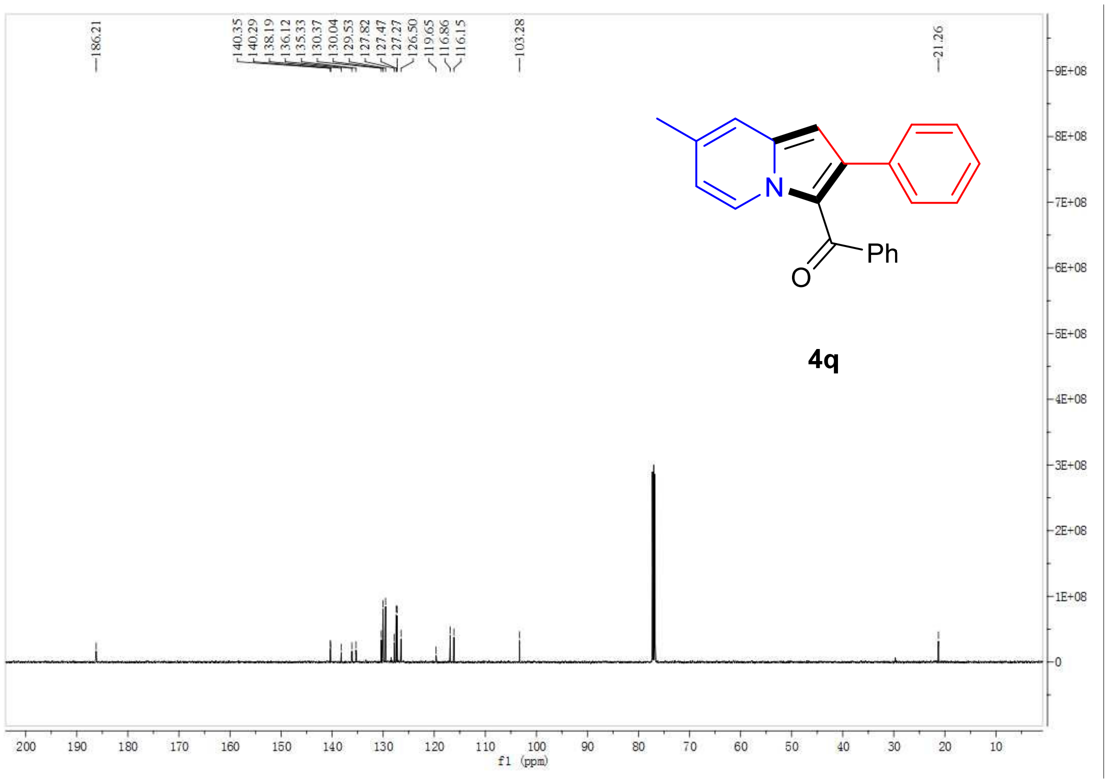
4q
f1 (ppm)

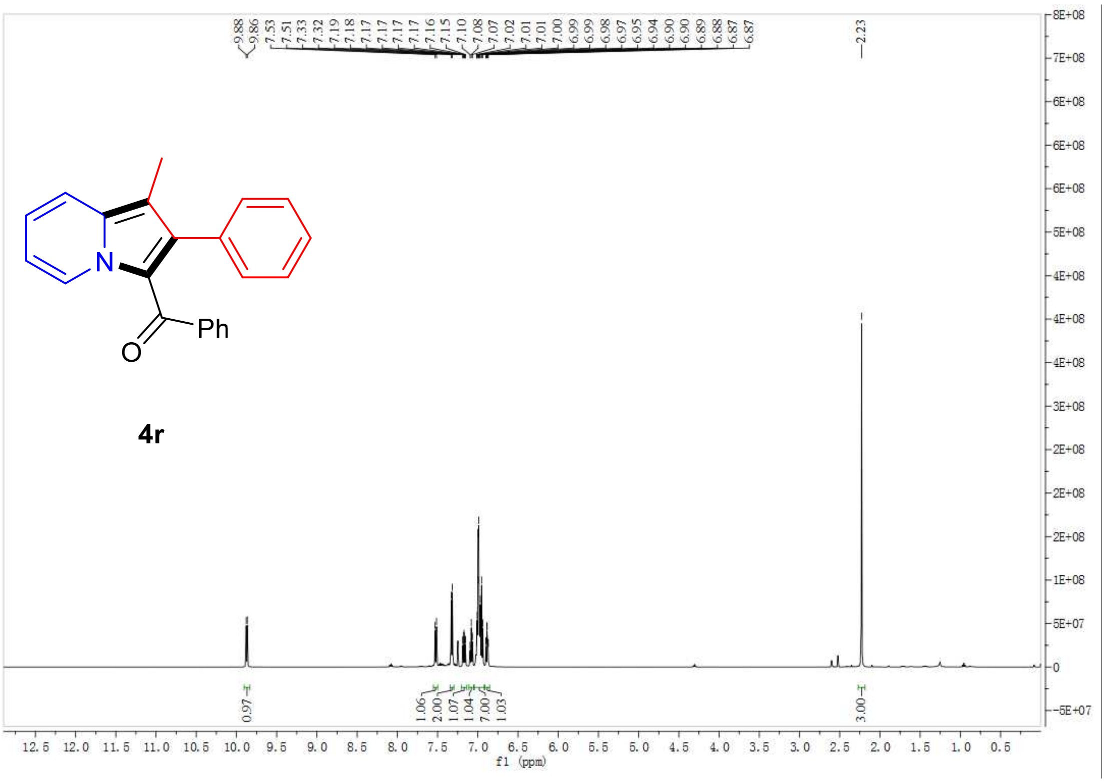
Ph
O
N
4r
9.88
9.86
7.53
7.51
7.33
7.32
7.19
7.18
7.17
7.17
7.17
7.17
7.16
7.15
7.10
7.08
7.07
7.02
7.01
7.01
7.00
6.99
6.99
6.98
6.97
6.95
6.94
6.90
6.90
6.89
6.88
6.87
6.87
2.23
0.97
1.06
2.00
1.07
1.04
7.00
1.03
3.00
f1 (ppm)

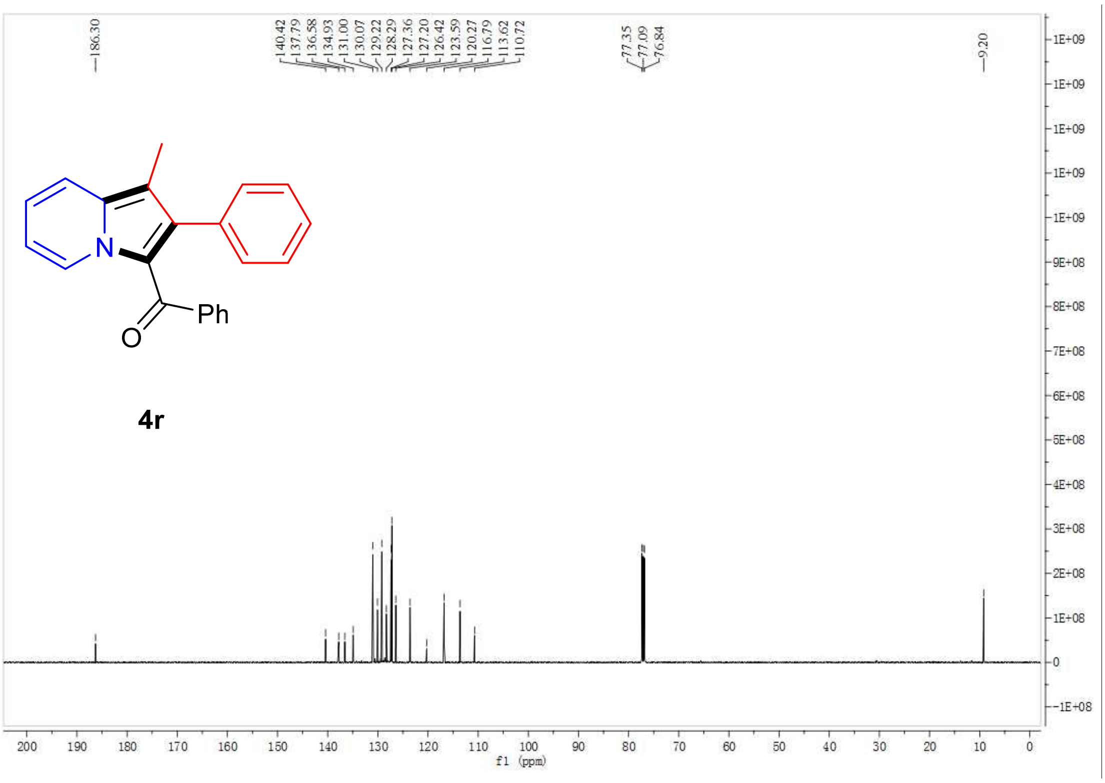
Ph
O
N
4r
186.30
140.42
137.79
136.58
134.93
131.00
130.07
129.22
128.29
127.36
127.20
126.42
123.59
120.27
116.79
113.62
110.72
77.35
77.09
76.84
9.20
f1 (ppm)

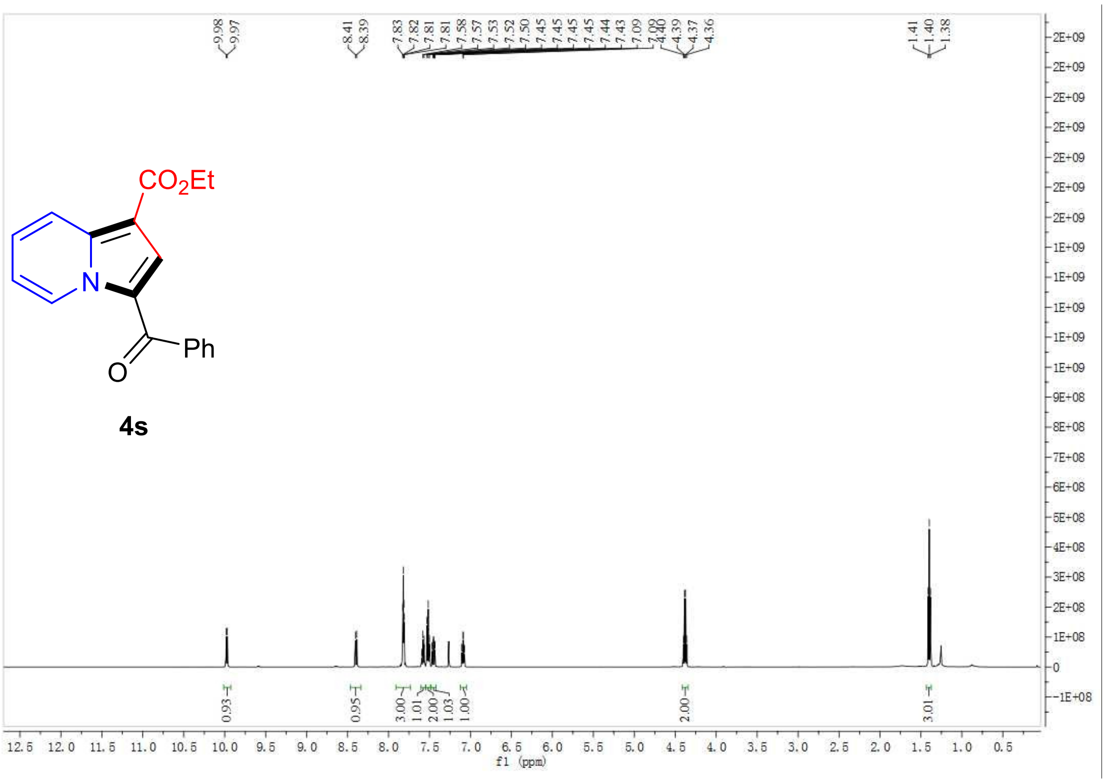

4s

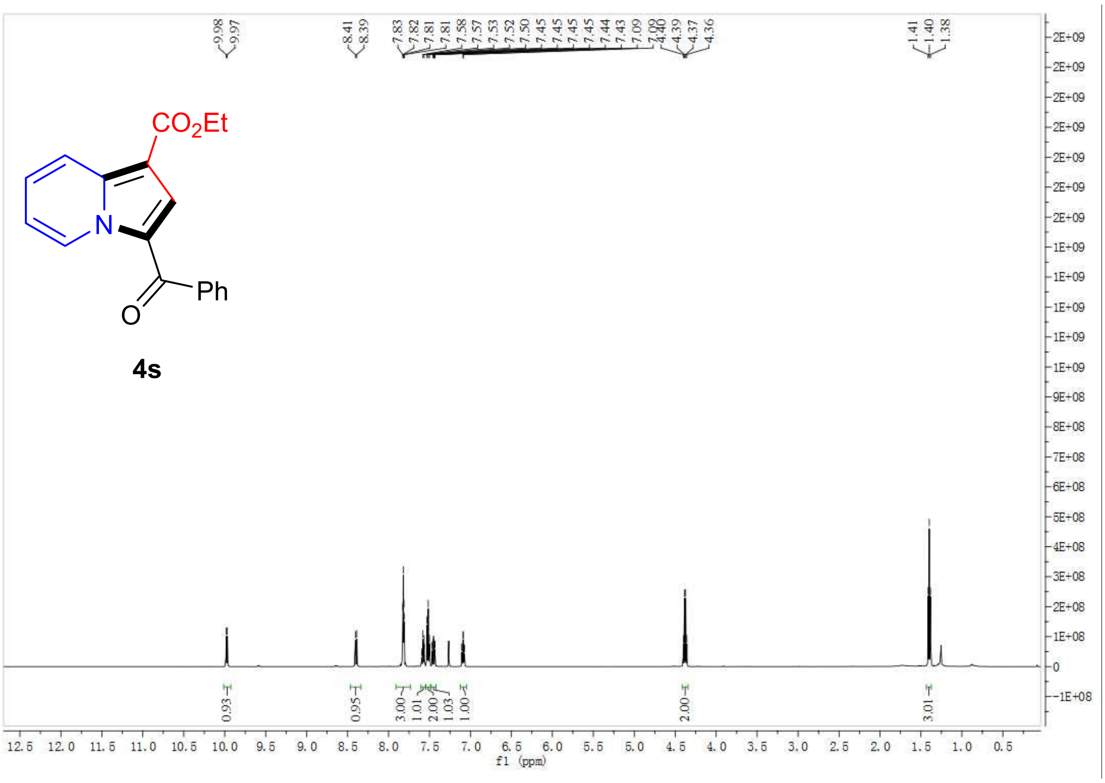

4s

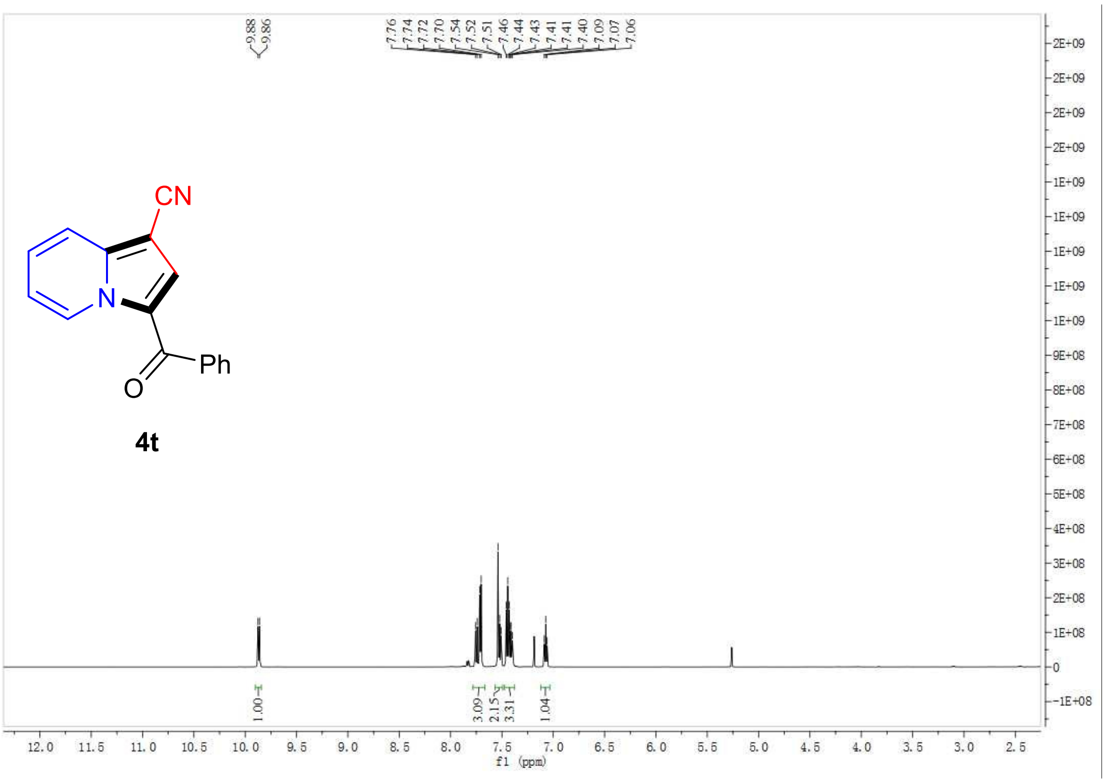

9.88
9.86
7.76
7.74
7.72
7.70
7.54
7.52
7.51
7.46
7.44
7.43
7.41
7.41
7.40
7.09
7.07
7.06
CN
N
Ph
O
4t
1.00
3.09
2.15
3.31
1.04
f1 (ppm)

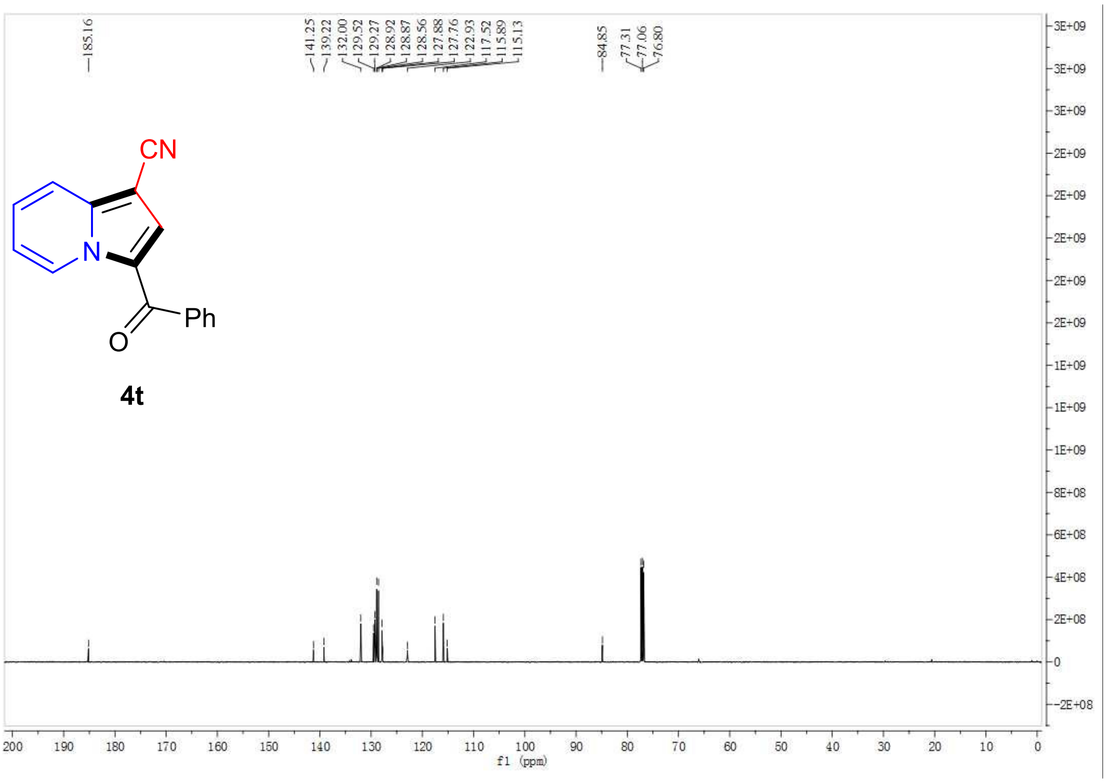

185.16
141.25
139.22
132.00
129.52
129.27
128.92
128.87
128.56
127.88
127.76
122.93
117.52
115.89
115.13
84.85
77.31
77.06
76.80
CN
N
Ph
O
4t
f1 (ppm)

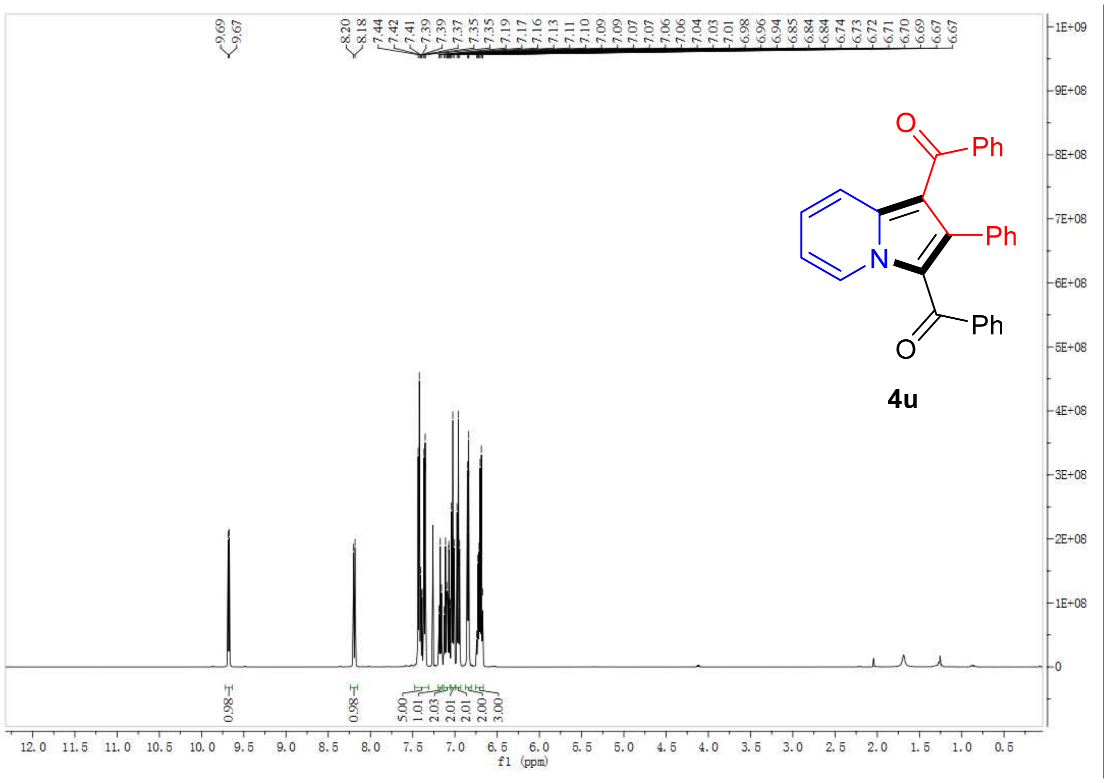

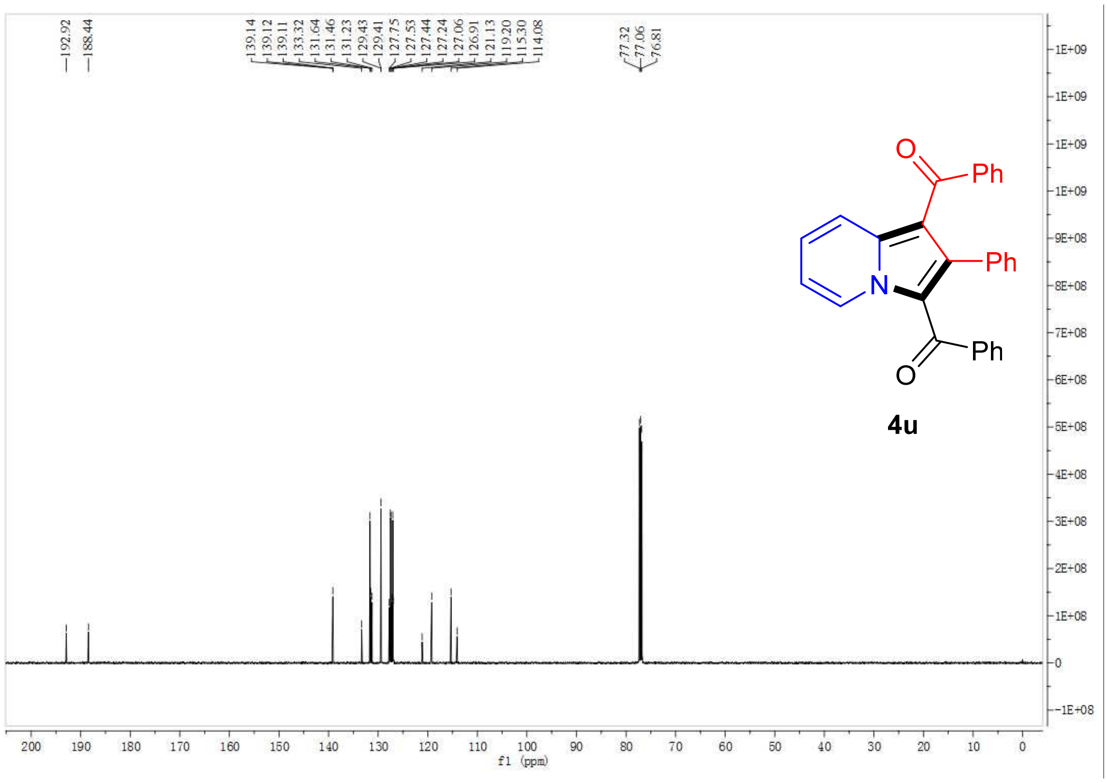

Supplement: Supplementary file 1 [file molecules-29-02061-s001.zip › molecules-2958040-supplementary.pdf]
